# Aberrant Excitatory–Inhibitory Synaptic Mechanisms in Entorhinal Cortex Microcircuits During the Pathogenesis of Alzheimer’s Disease

**DOI:** 10.1093/cercor/bhz016

**Published:** 2019-02-15

**Authors:** Alexandra L Petrache, Aarib Rajulawalla, Anqi Shi, Andrea Wetzel, Takashi Saito, Takaomi C Saido, Kirsten Harvey, Afia B Ali

**Affiliations:** 1 UCL School of Pharmacy, University College London, London, UK; 2 RIKEN Center for Brain Science, Wako-shi, Saitama, Japan

**Keywords:** Alzheimer’s disease, GABA_A_ receptor, interneurons, synapse, Wnt signaling

## Abstract

Synaptic dysfunction is widely proposed as an initial insult leading to the neurodegeneration observed in Alzheimer’s disease (AD). We hypothesize that the initial insult originates in the lateral entorhinal cortex (LEC) due to deficits in key interneuronal functions and synaptic signaling mechanisms, in particular, Wnt (Wingless/integrated). To investigate this hypothesis, we utilized the first knock-in mouse model of AD (*App*^*NL-F/NL-F*^), expressing a mutant form of human amyloid-β (Aβ) precursor protein. This model shows an age-dependent accumulation of Aβ, neuroinflammation, and neurodegeneration. Prior to the typical AD pathology, we showed a decrease in canonical Wnt signaling activity first affecting the LEC in combination with synaptic hyperexcitation and severely disrupted excitatory–inhibitory inputs onto principal cells. This synaptic imbalance was consistent with a reduction in the number of parvalbumin-containing (PV) interneurons, and a reduction in the somatic inhibitory axon terminals in the LEC compared with other cortical regions. However, targeting GABA_A_ receptors on PV cells using allosteric modulators, diazepam, zolpidem, or a nonbenzodiazepine, L-838,417 (modulator of α2/3 subunit-containing GABA_A_ receptors), restored the excitatory–inhibitory imbalance observed at principal cells in the LEC. These data support our hypothesis, providing a rationale for targeting the synaptic imbalance in the LEC for early stage therapeutic intervention to prevent neurodegeneration in AD.

## Introduction

The axis between the entorhinal cortex and the hippocampus, important for the formation and consolidation of memories, is thought to be the first brain region to be significantly affected in patients with Alzheimer’s disease (AD), characterized by synaptic loss leading to neurodegeneration and progressive cognitive deficits (Palop et al. 2006). Postmortem AD brains present with amyloid-β (Aβ) plaques, neurofibrillary tangles, dystrophic neurites, and signs of neuroinflammation, including astrocytosis and gliosis (Holtzman et al. 2011). Further to these contributors to the disease, deregulation of canonical Wingless/integrated (Wnt) signaling, important for synaptic maintenance, has long been proposed as a key contributor to neurodegeneration including AD pathogenesis (Inestrosa and Toledo 2008; Inestrosa and Arenas 2010; Toledo and Inestrosa 2010; Berwick and Harvey 2014).

One of the first changes observed during the early stages of AD pathogenesis is “hyperexcitability” in neuronal circuits. This is evidenced by imaging studies from preclinical and symptomatic AD patients during memory tasks (Buckner et al. 2005; O’Brien et al. 2010; Bero et al. 2012). It has been shown that the observed hyperexcitability is initiated in the lateral entorhinal cortex (LEC) before it spreads to other cortical regions (Khan et al. 2014). This abnormal hyperactivity is thought to be detrimental by causing Aβ release, spreading and accumulating during AD progression (Kamenetz et al. 2003; Busche et al. 2008; Cirrito et al. 2008; Yamamoto et al. 2015). This idea is further supported by studies reporting that a low dose of the antiepileptic drug levetiracetam can reduce hippocampal hyperactivity in humans and improve amnestic mild cognitive impairment (Bakker et al. 2012).

Although the precise neuronal circuits that are responsible for the hyperexcitability in AD patients observed from imaging studies remain largely unknown, it is generally accepted that glutamatergic principal pyramidal cells play a fundamental role in this neuronal hyperexcitation. The overall excitability of pyramidal cells is tuned by γ-aminobutyric acid (GABA)ergic inhibitory interneurons comprising ~20% of all neurones present in the cerebral cortex. As GABAergic cells are highly diverse in their properties and synapse on specific postsynaptic subcellular domains, (Maccaferri and Lacaille 2003; Markram et al. 2004; Ascoli et al. 2008; Klausberger and Somogyi 2008; Pelkey et al. 2017) even subtle disruption in interneuron behavior could unequivocally impact glutamatergic release, resulting in aberrant cortical excitation (Marin 2012; Verret et al. 2012; Palop and Mucke 2016; Khan et al. 2018).

Interestingly, fine regulatory mechanisms of Wnt signaling are crucial for synaptic maintenance that includes presynaptic neurotransmitter release, glutamate receptor trafficking and postsynaptic receptor clustering by postsynaptic density protein 95 (PSD-95) (Inestrosa and Arenas 2010; Cerpa et al. 2010, 2011; Park and Shen 2012). This signaling system has recently been shown to be important for the fine regulation of the entorhinal cortex–hippocampal circuitry (Oliva and Inestrosa 2015), and proposed to be critical for learning and memory-related synaptic plasticity (Jensen et al. 2012; Oliva et al. 2013a,b). However, it is unclear when neuronal hyperexcitability and Wnt signaling dysregulation occurs during the pathogenesis of AD.

The reported hyperexcitability in the brains of AD patients could be perceived as paradoxical, since several reports have shown that the inhibitory GABA receptor (GABA_A_) family is actually preserved in human brains of AD patients (Howell et al. 2000; Rissman et al. 2007), although the GABA_A_ receptors subtype that survives remains largely unclear. This receptor family is known to play a vital role in cognitive functions, including learning and memory through the actions of GABA-containing inhibitory interneurons. The prominent subclass of fast-spiking parvalbumin (PV)-containing (FS-PV) interneurons, namely, the basket cells targeting somatic and proximal dendrites of postsynaptic cells and chandelier cells targeting axon initial segments (Kawaguchi and Kubota 1993; Klausberger et al. 2005; Ascoli et al. 2008), accounts for ~40% of cortical GABAergic neurons (Rudy et al. 2011). FS-PV cells mediate inhibition via specific α1 and either α2 or α3 subunit-containing inhibitory GABA_A_ receptors found on postsynaptic principal cells (Ali and Thomson 2008). This GABA_A_ receptors, combination results in these cells showing activation in the γ frequency range in AD mice thereby reducing the spread of Aβ 1–40 and Aβ 1–42 isoforms before the onset of plaque formation or cognitive decline (Iaccarino et al. 2016). This suggests that the correct physiological function of FS-PV interneurons is important in preventing the spread of neurodegeneration in early stages of AD. The underlying mechanisms of this paradox and its significance for the expression of AD symptoms is not however, fully understood.

In our present study, we addressed this knowledge gap and deepened our understanding of synaptic dysfunction during AD progression using the first β-amyloid precursor protein (*App*) knock-in mouse model (*App*^*NL-F/NL-F*^). This novel model is unique in resembling human AD progression more accurately than *App* overexpression models, showing age-related Aβ pathology, memory impairment and neuroinflammation (Saito et al. 2014; Masuda et al. 2016; Sasaguri et al. 2017). We hypothesize that Wnt signaling dysregulation is correlated with disrupted activity of the major inhibitory PV interneuron microcircuitry, which results in hyperexcitability initiated in the LEC and that these pathomechanisms precede the formation of Aβ plaques and neuroinflammation in App knock-in mice. Therefore, we investigated the excitatory–inhibitory synaptic inputs in principal cells and Wnt signaling dysregulation in cortical regions including the LEC of the AD mouse model relative to wild-type controls. We further investigated whether pharmacological modulation of PV cell activity could rescue the aberrant synaptic hyperactivity in AD.

## Methods

### Animals

#### Experimental Animals

All of the procedures in the study were carried out in accordance with the British Home Office regulations under the Animal Scientific Procedure Act 1986, under the project license PPL: 7007558 held by the principal investigator, Dr Afia Ali. All procedures were approved by both internal and external UCL ethics committees, and in accordance with the ARRIVE guidelines for reporting experiments involving animals (McGrath et al. 2010). A total of 85 animals (disease model and wild-type) were used in this study. The animals had ad-libitum access to food and water and were reared in cages of maximum 5 inhabitants, with a day:night cycle of 12 h:12 h.

The knock-in *APP*^*NL-F/NL-F*^ AD mouse model was used for experiments (Saito et al. 2014). This particular mouse model was chosen because it follows the progression of human AD more faithfully. Since amyloid β-peptide (Aβ) plaque deposition is a key AD pathological hallmark, the model exhibits pathogenic Aβ accumulation whilst also maintaining biological amyloid precursor protein (APP) levels without overexpression artefacts. The *APP*^*NL-F*^ model consists of the introduction of 2 familial AD (FAD) mutations: KM670/671NL and I716F. The former, identified as the Swedish mutation, increases β-site cleavage of APP to produce elevated amounts of both Aβ_40_ and Aβ_42_, whereas the latter, known as the Beyreuther/Iberian mutation, promotes γ-site cleavage at C-terminal position 42, thereby increasing the Aβ_42_/Aβ_40_ ratio in favor of the more hydrophobic Aβ_42_ (Saito et al., 2014). Both features are key to the integrity of the disease phenotype. The transgenic line was crossed with C57BL/6 mice and the resulting heterozygous pairs were used for breeding, but excluded from experiments.

The knock in line was crossed with C57BL/6 mice and the resulting heterozygous pairs were used for breeding, but excluded from experiments. Only male *APP*^*NL-F/NL-F*^ and age-matched wild-type (C57BL/6) mice from the same breeding were used as control. *APP*^*NL-F/NL-F*^ and control mice were investigated at 3 different ages: grouped into 3 age groups: 1–2, 4–6, and 10–18 months.

Animals were genotyped via standard polymerase chain reaction using the following 4 primers: 5′-ATCTCGGAAGTGAAGATG-3′, 5′-TGTAGATGAGAACTTAAC-3′, 5′-ATCTCGGAAGTGAATCTA-3′, and 5′-CGTATAATGTATGCTATACGAAG-3′ as previously described (Saito et al. 2014).

#### Tissue Collection and Preparation

Mice were anesthetized by an intraperitoneal injection of 60 mg/kg phenobarbitone and perfused transcardially with artificial cerebrospinal fluid (ACSF) containing sucrose. The level of anesthesia was monitored using pedal, tail pinch reflexes, rate, depth, and pattern of respiration through observation and color of mucous membranes and skin. The ACSF comprised of (in mM) 248 sucrose, 3.3 KCl, 1.4 NaH_2_PO_4_, 2.5 CaCl_2_, 1.2 MgCl_2_, 25.5 NaHCO_3_, and 15 glucose, which was bubbled with 95% O_2_ and 5% CO_2_. The animals were then decapitated and the brain removed and parasagittal slices of cortex and hippocampus—300 μm thick—were cut in ice-cold standard ACSF using an automated vibratome (Leica, Germany). This standard ACSF contained (in mM): 121 NaCl, 2.5 KCl, 1.3 NaH_2_PO_4_, 2 CaCl_2_, 1 MgCl_2_, 20 glucose, and 26 NaHCO_3_, equilibrated with 95% O_2_ and 5% CO_2_. Slices were incubated in ACSF for 1 h at room temperature (20–23 °C) prior to recording. Brain slices were placed in a submerged chamber and superperfused with ACSF at a rate of 1–2 mL min^−1^ for electrophysiological recordings. For neuroanatomical studies, brains were immediately fixed after perfusion in 4% paraformaldehyde plus 0.2% picric acid in 0.1 M phosphate buffer (PB) for 24 h prior to sectioning.

#### Electrophysiology

Whole-cell somatic recordings were performed in LEC (or CA1 and layer 2/3 of the neocortex) pyramidal cells and interneurons. Patch electrodes with resistances of 8–11 MΩ were made from filamented borosilicate glass capillaries (Harvard Apparatus, UK) and filled with a solution containing (in mM): 134 K gluconate, 10 HEPES, 10 phosphocreatine, 2 Na_2_ATP, 0.2 Na_2_GTP, and 0.2% w/v biocytin. Neurons were selected for recording based on the shape of their soma using video microscopy under near infrared differential interference contrast illumination, and further characterized by their electrophysiological properties obtained from injecting a series of 500 ms depolarizing and hyperpolarizing current pulses. Action potential parameters were measured from responses to depolarizing current steps (+50 to 150 pA, 500 ms), which induced a single or a trains of action potentials. The input resistance and membrane time constant were determined from voltage changes in response to hyperpolarizing current steps (−100 pA, 500 ms).

Spontaneous postsynaptic potentials were recorded from passive membrane responses and mixed spontaneous excitatory postsynaptic potentials (sEPSPs) and spontaneous inhibitory postsynaptic potentials (sIPSPs) were collected in 60 s frame samples, repeated at 0.33 Hz. Recordings were carried out under the current clamp mode of operation (NPI SEC 05LX amplifier; NPI electronics, Germany), low pass filtered at 2 KHz and digitized at 5 KHz using a CED 1401 interface (Cambridge Electronic Design, UK). Input resistance was monitored throughout experiments by means of a hyperpolarizing current step (−0.001 nA, 10 ms). Signal (Cambridge Electronic Design, UK) was used to acquire recordings and generate current steps. The average amplitudes of spontaneous events and their frequency was measured manually from single sweep data sets of 60 s recordings, including a total sweep range of 30–50 frames (i.e., 30–50 min of recording). For in vitro pharmacological studies, the GABA_A_ receptor allosteric modulators—diazepam, zolpidem, or L-838,417 (0.5–1 μM, Tocris Bioscience, UK), the GABA_A_ receptor antagonist GABAzine (SR95531 hydrobromide), and tetrodotoxin (TTX) were bath-applied. Average data points after drug application were obtained after steady-state responses were attained with the drugs, which was ~15–20 min after onset of the bath-application.

#### Neuroanatomical Procedures and Analysis

Parasagittal sections containing the entorhinal cortex, cerebral cortex, and hippocampus were sectioned at 100 μm-thickness using a vibratome (Vibroslice, Camden Instrument, Loughborough, UK), and placed in a 24-well plate containing 10% PB. Each experiment consisted of slices from wild-type and *APP*^*NL-F/NL-F*^ age-matched mice and kept in separate 24-well plates. The sections per brain were randomly allocated to the antibody and procedure, but all sections underwent identical protocols for either immunofluorescence or immunoperoxidase procedures. Prior to these specific procedures, all sections were washed in 0.1% Triton X-100 in Tris-buffered saline (TBS-T), followed by incubation in 1% hydrogen peroxide aqueous solution for 30 min. After further rinses in TBS-T, sections were incubated in phosphate buffer saline (PBS) containing 10% normal goat serum (Sigma-Aldrich, USA) for 1 h at room temperature. This followed incubation in the specific primary antibodies to target the desired proteins shown in Table 1.

**Table 1:** Antibodies used in immunohistochemistry experiments.

Immunofluorescence primary antibodies			
Antibody target	Company	Targeted species	Dilution
Parvalbumin	SWANT	Rabbit	1:1000
GAD67	Millipore	Mouse	1:2000
CD68	BioRad	Goat	1:500
GFAP	Agilent (Dako)	Rabbit	1:500
VGlut1	Millipore	Goat	1:2500
GAT-1	Millipore	Goat	1:500
CamKII-α	Cell Signaling Technology	Mouse	1:100
Immunofluorescence secondary antibodies			
Antibody	Company	Targeted species	Dilution
Texas Red	Thermo-Scientific	Rabbit	1:500
FITC	Sigma-Aldrich	Mouse	1:875
Alexa 488	abcam	Rabbit	1:1000
Alexa 568	Molecular Probes	Goat	1:500
DAPI	Sigma-Aldrich	Multiple	1:1000
Immunoperoxidase primary antibodies			
Antibody target	Company	Targeted species	Dilution
Parvalbumin	SWANT	Rabbit	1:5000
APP695	Thermo-Fisher	Mouse	1:10 000
CD68	BioRad	Goat	1:8000
GFAP	Agilant (Dako)	Rabbit	1:2000
Immunoperoxidase secondary antibodies			
Antibody	Company	Targeted species	Dilution
Biotinylated	Vector	Rabbit, Mouse, Rat	1:500

##### Immunofluorescence procedures, confocal image acquisition and analysis

For example, the anti-PV and anti-GAD67 primary antibodies were both added to the same wells to allow for colocalization assessments to be performed, whilst the anti-APP695, anti-CD68 and anti-GFAP primary antibodies were added to independent wells.

After incubation for 48 h in primary antibody on a platform shaker at a temperature at 4 °C, and a further 3 washes in TBS-T, the sections were incubated in the appropriate secondary antibodies for 3 h (fluorophores for immunofluorescence are shown in Table 1). The sections were then washed (3 × 10 min 0.3% TBS-T per well) and slices allocated for either CD68 or GFAP- selective immunofluorescence staining were then incubated further with DAPI (1:1000 dilution) for 15 min. After further washes, the slices were then mounted on glass slides using the antifade mounting medium, Vectarshield (Vector Lab. UK) ready for confocal microscopy.

The acquisition of fluorescence images was obtained using a LSM 710 confocal microscope which processed images using the Zen Black 2009 ZEISS software. An overview image (Tile-Scan) was taken of each whole brain slice using the ×10 lens, and then 3D images (*Z*-stack) were taken at 6 different positions within each brain section: the dorsal entorhinal cortex 1), ventral entorhinal cortex 2), layers 2/3 of the somatosensory neocortex 3), CA1 4), CA2 5) and CA3 regions 6), using the ×20 lens. The first and last 10 μm were discarded from each section to prevent repeated capture of the same cell.

The mean intensity and standard deviation (SD) of the light emitted by the fluorophores within a maximum projection of the *Z*-stacks was calculated using the confocal image processing software ZEN. To distinguish PV-expressing cells from any background fluorescence, a threshold was determined; any emission that exceeded a value of the mean intensity plus twice the standard deviation (e.g., PV or GAD67-positive interneuron = mean intensity + [2 × SD mean intensity]). Colocalization was confirmed when fluorophores marking both PV and GAD67 were at an intensity that exceeded the threshold. Somata that exceeded the threshold were counted in the individual levels of the *Z*-stack; any positive cells that appeared on consecutive levels of the *Z*-stack were considered to be the same cell and were not recounted. The total positive cell number of each *Z*-stack was then divided by the volume of the *Z*-stack (4.99 × 10^−2^ mm^3^) to determine the density of PV + GAD67-coexpressing interneurons in each of the areas of interest.

Cell counts for each individual segment, were performed manually by circling each cell with the intent of avoiding counting the same cell twice. The total volume of each segment was calculated to allow for the conversion of cell counts to signify neuronal cell density (number of cells/mm^3^).

##### Immunoperoxidase procedure and analysis

After washes in TBS-T, the sections were then incubated in secondary biotinylated antibodies (see Table 1 for individual antibody detail). Postincubation with secondary fluorescent antibody and after washes in TBS-T, there was a further incubation in avidin–biotin–horseradish peroxidase complex (ABC; Vector Laboratories, UK) solution, for 2 h at room temperature. The sections were then washed further in TBS-T, and processed with 3–3-diaminobenzidine (DAB), and subsequently dehydrated and mounted (Khan et al. 2018).

The darkness density of slices was measured using the Fiji imaging package. DAB-stained pictures were taken under 10× light microscope and kept consistent background. Pictures were processed by color deconvolution and “H DAB,” and “Mean Gray Value” was used to measure the darkness density. Mean gray values were normalized into optical density numbers by the formula: OD = log (max intensity/mean intensity), where max intensity = 255 for 8-bit images.

##### Recovery of biocytin labeled-cells postelectrophysiological recordings and morphometric analysis

After electrophysiological recordings of pyramidal cells in the entorhinal cortex, slices were fixed in 4% paraformaldehyde plus 0.2% picric acid in 0.1 M PB for 24 h and then re-sectioned at 70 μm. Slices were incubated in ABC overnight at 4 °C, followed by the above DAB protocol. Anatomically recovered cells were reconstructed manually from consecutive slices at 100× objective under a Leica DMR microscope with an attached drawing tube. Images from the consecutive slices were digitally superimposed so as to be analyzed using Sholl analysis in ImageJ (version 1.49, RBS, Maryland, USA). Only neurons that were fully stained and had intact dendritic arbors were included in the study. The Sholl analysis was carried out with a radius step size of 10 μm and the area under the curve was regarded as a determinant of dendritic complexity, meaning that any deviations of this feature represented morphological alterations (Ristanovic et al. 2006).

### Canonical Wnt Signaling Analysis via Immunoblotting

Brains were collected from *App*^*NL-F/NL-F*^ knock-in mice and age-matched wild-type controls, micro dissected into cortex, entorhinal cortex and cerebellum and directly transferred to ice. All following steps were performed on ice. Brain lysates were generated in lysis buffer composed of 5 mM MgCl_2_, 150 mM NaCl, 50 mM Tris, pH 7.5, 1% (v/v) Igepal, supplemented with 1× complete protease inhibitor cocktail (Roche) and 1:100 phosphatase inhibitor cocktail (Pierce) using a dounce homogenizer (Merck). Lysates were cleared through a 10 min centrifugation step at 14 000 *g* and protein concentration was determined with the bicinchoninic acid (BCA) assay (Pierce). Equal protein amounts were analyzed by SDS Polyacrylamide gel electrophoresis (SDS-PAGE) and immunoblotting. Thereafter, 4× LDS sample loading buffer and 10× sample reducing agent (Invitrogen) were added to the samples followed by incubation for 10 min at 98 °C. The denatured samples were loaded onto a 4–12% (w/v) BisTris precast gel (Invitrogen). Proteins were blotted on polyvinylidine fluoride (PVDF) membranes (Millipore), blocked with 5% (w/v) nonfat dry milk in Tris-buffered saline plus 0.1% (v/v) Tween 20 (TBS-T) for 30 min and labeled using the following primary antibodies: active β-catenin (clone 8E7, Millipore), full-length β-Amyloid (clone 6E10, BioLegend) and β-actin (Sigma) in blocking buffer overnight at 4 °C. Membranes were washed 3 times in TBS-T the following morning, prior to incubation in HRP-conjugated secondary antibodies (Santa Cruz) in blocking buffer for 2 h at room temperature on a rocking shaker. Washing was repeated and the signal detected using SuperSignal West Pico Chemiluminescent Substrate or SuperSignal West Femto Chemiluminescent Substrate (Pierce) and a Syngene GeneGnome Imaging system. For the quantification, ImageJ software was employed. Please refer to supplementary files for further Western blots.

### Statistics

A one-way analysis of variance (ANOVA) was used to determine whether there were any significant differences between the different experimental cohorts. If there was a difference, the Student *t*-test (2-tailed, unpaired) was used to compare *App*^*NL-F/NL-F*^ animals to wild-type counterparts separately at the age group of interest. *P*-values below 0.05 were deemed significant. All figures displaying error bars represent the standard deviation from the mean, unless stated otherwise. The bar graphs display cohort means.

The “*n*” are given as the total number of observations (cells) and the number of animals used, unless otherwise stated.

## Results

### The App^NL-F/NL-F^ Mouse Model Shows Time-Dependent Accumulation of Aβ and Neuroinflammation Associated With a Steady Decline of Principal Cell Density

In the *App*^*NL-F/NL-F*^ mouse model, we observed age-dependent phenotypic changes of AD, including neuroinflammation indicated by astrocytosis, microgliosis and the progressive Aβ accumulation and deposition leading to plaque formation, which advocates progressive neurodegeneration, accurately recapitulating progression in AD patients. These data are also consistent with the study by Saito et al. (2014).

**Figure 1.**
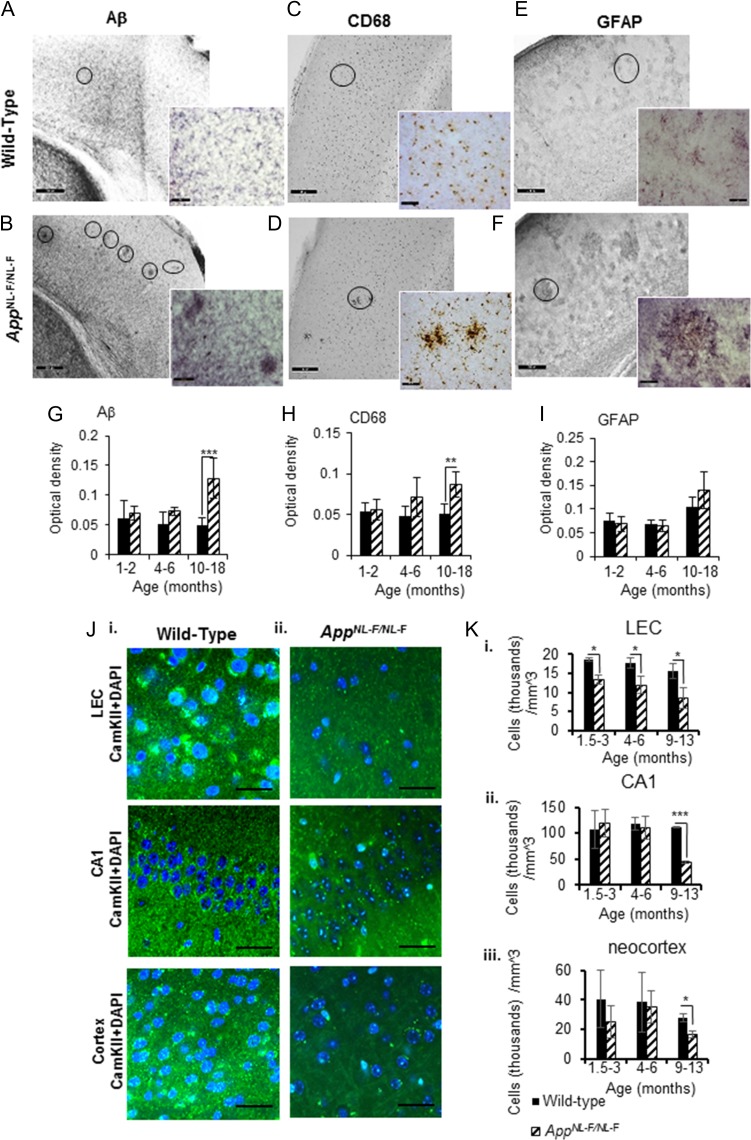
The first knock-in AD mouse model, App^NL-F/NL-F^ shows an age-dependent accumulation of Aβ pathology, neuroinflammation and neurodegeneration of principal cells. (*A*) and (*B*) Immunoperoxidase labeling of Aβ in aged wild-type and App^NL-F/NL-F^ brains. Photographs taken with light microscope at 20× magnification; inserts illustrate photographs taken at 40× magnification. Circles show Aβ deposits. (*C*–*F*) Similarly, CD68 and GFAP labeling indicating microglia and reactive astrocytes respectively, in aged wild-type and App^NL-F/NL-F^ brains. The presence of strong Aβ aggregation in the AD mouse model, which correlates with increased inflammatory markers, is apparent (scale bar, 50 μm, enlarged images, scale bar, 20 μm). Circles indicate microglia and reactive astrocytes, respectively. (*G*–*I*) Graphs illustrating an age-dependent increase in Aβ, CD68, and GFAP levels in the 3 different age windows investigated in wild-type and *App*^*NL-F/NL-F*^. *App*^*NL-F/NL-F*^ mice (10–18 months) demonstrated a significant increase in Aβ and CD68 level compared with wild-type aged animals, or the other 2 age cohorts (1–2 months, 4–6 months). Results are expressed as mean ± SD (**P < 0.01, ***P < 0.001; 2-tailed student *t*-test). (*J*i-ii) Confocal imaging experiments using antibody to selectively label the expression of calcium/calmodulin-dependent protein kinase II (CamKII) expressed in principal excitatory cells in 3 cortical regions: LEC, CA1, and layer 2/3 of the neocortex. There is gradual decline in DAPI colocalised with CamKII (secondary antibody Alexa 488, green), suggesting neurodegeneration of principal cells in the *App*^*NL-F/NL-F*^ mouse model. Panels show representative confocal images taken at X63 magnification of tissue stained with CamKII and DAPI in wild-type animals at 13 months and in age-matched *App*^*NL-F/NL-F*^ mouse brains (scale bar = 20 mm). (*K*i-iii) Pyramidal cell density was counted from collapsed confocal *Z*-stacks taken at ×20 magnification. There is significant reduction in pyramidal cell density in the lateral entorhinal cortex in the *App*^*NL-F/NL-F*^ animals compared with age-matched wild-type mice at all 3 ages investigated, which reduced significantly, (*P < 0.05, ***P < 0.001, 2-tailed Student *t*-test comparing *App*^*NL-F/NL-F*^ animals to wild-type counterparts separately at each of the 3 age groups investigated, *n* = 5 animals per cohort).

There was a higher level of Aβ_42_/Aβ_40_ associated in the *App*^*NL-F/NL-F*^ mouse model at 10–18 months as opposed to their age-matched wild-type control mice (Fig. 1*A*,*B*,*G*). This accumulation of Aβ protein plaques is known to be initiated in the LEC and layer 2/3 of the neocortex border ((Braak and Braak 1991; Holtzman et al. 2011), which is consistent with our study showing an increase of Aβ protein plaques in the LEC of *App*^*NL-F/NL-F*^ mice by, 12.64 ± 2.21%, 44.95 ± 4.29% and 286.61 ± 74.13% of control mice at 1–2, 4–6, and 10–18 months, respectively, *P* < 0.01, *n* = 6 animals).

Immunoreactivity to the neuroinflammatory markers cluster of differentiation 68 protein (CD68) and glial fibrillary acidic protein (GFAP) were also shown to be differentially expressed in the wild-type mice age-matched to *App*^*NL-F/NL-F*^ mice. An age-dependent, gradual increase in CD68-positive glial cells in the LEC of *App*^*NL-F/NL-F*^ mice was also observed (increase by, 4.32 ± 0.98% and by, 48.08 ± 16.11%, and 71.29 ± 12.72% of control mice at 1–2, 4–6, and 10–18 months, respectively), however these differences were only significant at 10–18 months (*P* < 0.01, *n* = 6 animals, Fig. 1*C*,*D*,*H*).

A slight decrease of GFAP-positive reactive astrocytes in the LEC was observed in the *App*^*NL-F/NL-F*^ mouse model at 1–2 and 4–6 months compared with wild-type control mice (decreased by, 5.80 ± 1.21%, 6.38 ± 1.29% of control wild-type mice at 1–2 and 4–6 months, respectively); however, a significant accumulation of active astrocytes was seen in APP^*NL-F/NL-F*^ mice at 10–18 months (31.50 ± 8.74% of control mice, *P* < 0.07, *n* = 6 animals) (Fig. 1*E*,*F*,*I*).

Figure 1 illustrates analysis from results obtained from immunoperoxidase procedures, however, these observations were consistent with complementary immunofluorescence labeling.

Furthermore, to investigate whether principal pyramidal cells showed neurodegeneration in the *App*^*NL-F/NL-F*^ mouse model, we performed immunohistochemistry using antibody against CaMKII-α which is expressed in principal cells (Wang et al. 2013), and costained the tissue with DAPI (nuclei staining). An age-dependent decline in pyramidal cell density in CA1 and LEC was observed, suggesting a physiological progression of neurodegeneration in the *App*^*NL-F/NL-F*^ mouse model of AD (Fig. 1*J*,*K*). Analysis of confocal imaging *Z*-stacks revealed that in the LEC, there was a significant decline in pyramidal cells in the *App*^*NL-F/NL-F*^ animals compared with age-matched wild-type mice at all 3 age windows investigated. At 1–2 months, a reduction of 28.72 ± 2.63%, at 4–6 months, a reduction of 32.11 ± 5.84%, and at 10–18 months, a reduction of 45.07 ± 14.71% was observed (these data were all significantly different from the control, *P* < 0.05, *n* = 5 animals per cohort). In the CA1 and neocortex, there was also a significant reduction in the expression of CaMKII-α expressed in pyramidal cells costained with DAPI in the *App*^*NL-F/NL-F*^ mouse brains compared with the healthy age-matched controls in the age window of 10–18 months, showing a reduction of 60.51 ± 3.98% (*P* < 0.001, *n* = 4 animals per group) in CA1 and 40.94 ± 6.23% (*P* < 0.05, *n* = 4 animals per group), in the neocortex. The reduction in the number of cells and fragmented staining also associated with a shrinkage of cells, were characteristic signs of cellular neurodegeneration in the cortical regions studied in the *App*^*NL-F/NL-F*^ model.

### Time-Dependent Wnt Signaling Changes in the *App^NL-F/NL-F^* Mouse Model of AD

**Figure 2.**
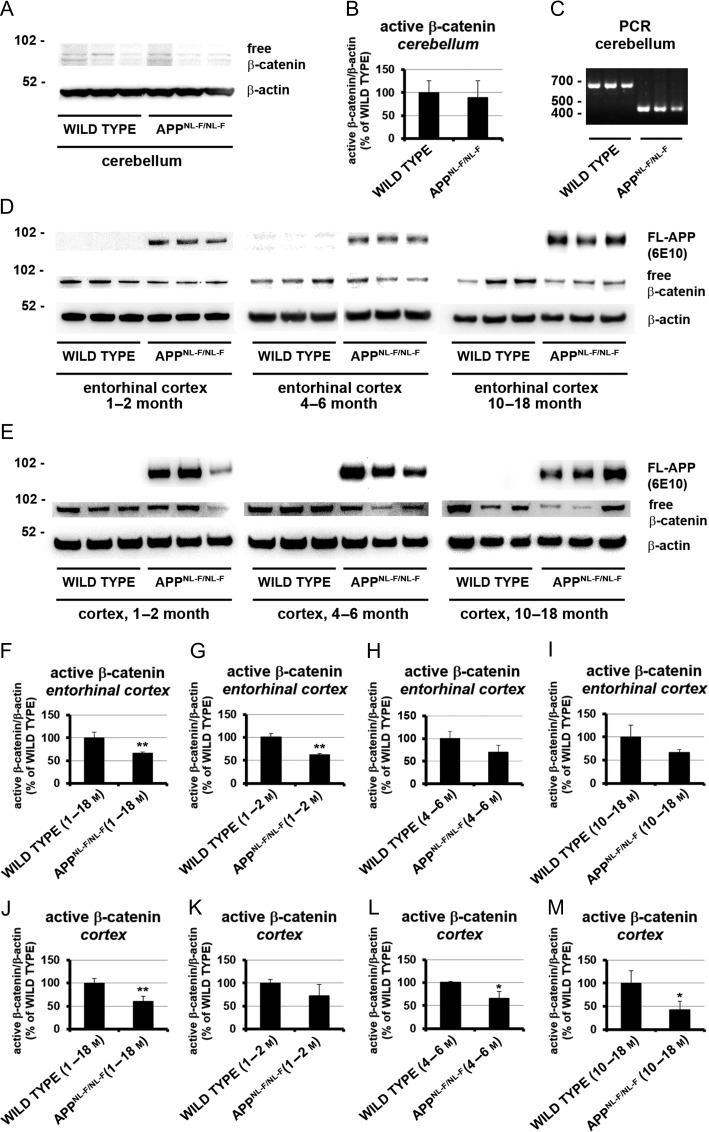
*T*issue and age-dependent Wnt signaling analysis of wild type and *App*^*NL-F/NL-F*^*knock-in mice*. Equal amounts of protein lysates from different brain regions were analyzed from 1 to 18 months old wild-type and *App*^*NL-F/NL-F*^ knock-in mice via immunoblotting. (*A*, *D*, and *E*) Representative Western blot analysis showing detection of active β-catenin in lysates from the cerebellum (*A*), entorhinal cortex (*D*), and cortex (*E*). Protein detection of human full-length APP (*D* and *E*) and mouse β-actin (*A*, *D*, and *E*) served as control. (*C*) Representative amplicon of 700 bp for wild type and 400 bp for *App*^*NL-F/NL-F*^ knock-in mice, confirming the genotype of animals used in (A) via polymerase chain reaction using genomic DNA. (*B*, *F*–*M*) Mean signal changes of active β-catenin in cerebellum (*B*), entorhinal cortex (*F*–*I*) and cortex (*J*–*M*). Western blot labeling showing age-dependent significant reduction of active β-catenin in the cortex of *App*^*NL-F/NL-F*^ knock-in mice compared with wild-type mice. Signals were divided through β-actin and normalized to wild-type (100%) represented as mean percentage ± SEM (*P < 0.05; **P < 0.01, *n* = 3–9).

Decreased canonical Wnt signaling has previously been suggested to play a role in AD pathogenesis in patients and has also been observed in other AD animal models (Inestrosa and Varela-Nallar 2014; Jin et al. 2017; Dengler-Crish et al. 2018; Huang et al. 2018; Tapia-Rojas and Inestrosa 2018a, b). Our aim was to investigate these findings in the *App*^*NL-F/NL-F*^ mouse model. In addition, we asked the question of when and in what brain areas, changes in canonical Wnt signaling can first be observed during pathogenesis. The entorhinal cortex is one of the earliest and most affected by the neurodegenerative disease process, whereas the cerebellum is rarely affected. Therefore, we investigated canonical Wnt signaling activity in the cortex, entorhinal cortex and cerebellum of *App*^*NL-F/NL-F*^ knock-in mice in comparison to wild-type littermate controls using an antibody against free dephosphorylated ß-catenin. We found an overall statistically significant decrease of canonical Wnt signaling activity in the cortex and entorhinal cortex to 60 ± 12% (*P* < 0.01) and 66 ± 3% (*P* < 0.01) respectively but no significant change in the cerebellum of *App*^*NL-F/NL-F*^ knock-in animals (Fig. 2, Supplementary Figs S1–S7). The decrease of canonical Wnt signaling activity was already evident from 1–2 months of age in the entorhinal cortex (62 ± 3%, *P* < 0.01) and from 4–6 months in the remaining cortex (66 ± 15%, *P* < 0.05). In the cortex we observed a decrease of canonical Wnt signaling activity over time from 1–2 months (72 ± 15%, not statistically significant), 4–6 months (66 ± 15%, *P* < 0.05) to 12–14 months (42 ± 19%, *P* < 0.05) old animals suggesting a progression of the signaling defect in a special and/or temporal manner. As the Wnt signaling dysfunction is first observed in the entorhinal cortex but seems to remain at a similar level in this area over time, a spread of the canonical Wnt signaling dysfunction throughout the cortex starting from the entorhinal cortex is suggested. Our data also show that the first observed decrease in Wnt signaling activity in the entorhinal cortex and cortex precedes the above observed gliosis and Aβ pathology in the *App*^*NL-F/NL-F*^ model (Fig. 1). Observed Wnt signaling activity is at its lowest point when AD pathology becomes visible in brain slices.

### Distorted Excitatory–Inhibitory Synaptic Activity in the LEC Preceding Hallmarks of AD

To determine whether the decrease of Wnt signaling activity was correlated with early stage synaptic impairment, we investigated whether the excitatory and inhibitory synaptic inputs received by principal pyramidal cells were impaired in the *App*^*NL-F/NL-F*^ model of AD. To examine the network effect at these cells, we first examined the presynaptic action potential-dependent synaptic release and recorded sEPSPs and sIPSPs from the 3 different age cohorts in age-matched wild-type and *App*^*NL-F/NL-F*^ mice in the LEC, CA1, and neocortex. This was then followed by recording miniature postsynaptic events (mEPSPs and mIPSPs) in the presence of the sodium channel blocker tetrodotoxin (TTX), which reveals action potential-independent mechanisms of the neurotransmitter release machinery.

**Figure 3.**
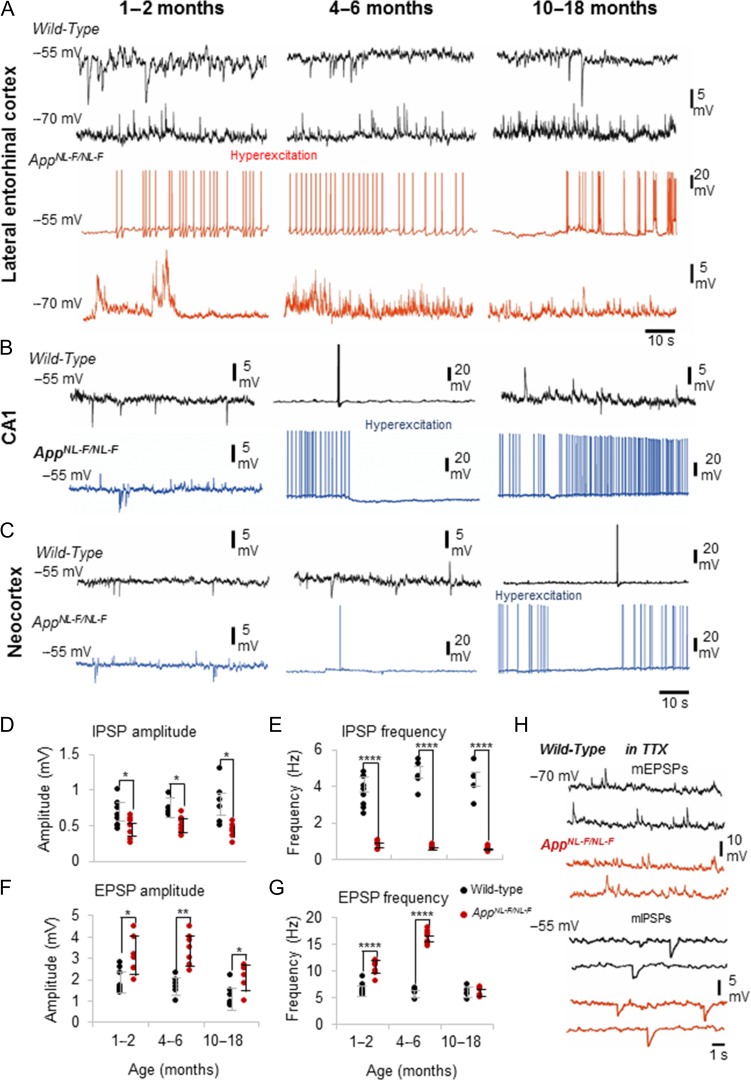
Persistent synaptic hyperexcitation and diminished inhibition at principal pyramidal cells in *App*^*NL-F/NL-F*^ mouse model of AD. Intracellular recordings of spontaneous response of layer 2 LEC neurons recorded using whole-cell patch clamp electrodes at membrane potentials held at −55 and −70 mV (in current clamp). (*A*) Recordings from wild-type mice at 1–2 months, 4–6 months, and 10–18 months at −70 and −55 mV. Similar recordings from age-matched *App*^*NL-F/NL-F*^ mice are shown in red. The principal, pyramidal cells recorded in wild-type mice showed less spontaneous firing and subthreshold oscillation compared with *App*^*NL-F/NL-F*^ mice when held at firing threshold of −55 mV. Furthermore, the wild-type mice showed inward inhibitory current events as expected at all ages investigated. By contrast, similar neurons recorded in AD mice resulted in oscillation between subthreshold and spontaneous action potential discharge at firing threshold of −55 mV, which was seen to diminish from 10 months onwards. These results suggest impaired spontaneous excitation and inhibition and an increasing state of hyperexcitability upon AD progression until 10–18 months. Scale bars on the right refer to all 3 data sets. (*B* and *C*) Similar events recorded from pyramidal cells in CA1 and in the layer 2/3 of the neocortex (somatosensory region) in wild-type (black traces) and age-matched *App*^*NL-F/NL-F*^ mice (blue traces). Hyperexcitation was *not* apparent in these excitatory cells in the *App*^*NL-F/NL-F*^ younger mice cohorts in CA1 or neocortex, as this developed later in contrast to the LEC. (*D* and *E*), sIPSP amplitudes and frequency recorded at −55 mV in wild-type and age-matched *App*^*NL-F/NL-F*^ mice. (*F* and *G*) sEPSP amplitudes and frequency recorded at −70 mV in wild-type and age-matched App^*NL-F/NL-F*^ mice. sEPSP amplitude and frequency were significantly higher in the *App*^*NL-F/NL-F*^ mouse model until 10–18 months, and correlated with a significantly reduced amplitude and frequency of sIPSPs received by pyramidal cells in the *App*^*NL-F/NL-F*^ mouse model (*P < 0.05; **P < 0.01, ***P < 0.001, ****P < 0.0001, *n* = 7 animals per cohort). (*H*) Synaptic events recorded in the presence of TTX, illustrating example traces of miniature, mEPSPs and mIPSPs in wild-type and age matched App^*NL-F/NL-F*^ mice.

The vast majority of pyramidal cells studied in layer 2 of the LEC (~90%) showed impaired excitatory and inhibitory properties recorded in the youngest cohort of 1–2 months of *App*^*NL-F/NL-F*^ mice (Fig. 3*A*). Pyramidal cells recorded in the CA1 region and neocortex did not show hyperexcitability in the *App*^*NL-F/NL-F*^ mice at 1–2 months and there was no significant different between the synaptic responses recorded in age-matched wild-type mice (20–30 cells recorded in CA1 and neocortex, *n* = 15 animals). However, in the *App*^*NL-F/NL-F*^ mouse model, an age-dependent spread of the hyperexcitability was observed; CA1 pyramidal cells exhibited hyperexcitability between 4 and 6 months, while neocortical pyramidal cells exhibited it at a later stage of 10 months onwards (Fig. 3*B*,*C*).

Due to the apparent aberrant hyperexcitability evident at 1–2 months in the LEC, we focused on investigating the synaptic changes in more detail here. We found that in the LEC, GABA_A_ receptor mediated sIPSPs recorded at −55 mV membrane potential from all 3 age cohorts of *App*^*NL-F/NL-F*^ mice showed a significant decrease in amplitude and frequency. The mean amplitude of sIPSPs decreased by 32 ± 9%, 32 ± 9%, 48 ± 38% of control mice for 1–2, 4–6, and 10–18 months, respectively (*P* < 0.01, *n =* 12 cells, *n* = 7 animals). The frequency of sIPSPs was also consistently lower in AD mice by 79 ± 25%, 85 ± 16%, 87 ± 18% of control mice for 1–2, 4–6, and 10–18 months, respectively (all significantly different *P* < 0.0001, *n =* 10 cells, *n* = 7 animals) (Fig. 3*A*,*D*,*E*). However, in contrast, both sEPSP amplitude and frequency were significantly *higher* in 1–2 months and 4–6 months aged *App*^*NL-F/NL-F*^ mice, but then reduced at 10–18 months. The sEPSP amplitudes were significantly higher in the *App*^*NL-F/NL-F*^ mice, by 70 ± 3%, 100 ± 22%, and 68 ± 29% of control mice at 1–2, 4–6, and 10–18 months, respectively (all significantly different *P* < 0.05, *n =* 12 cells, *n* = 7 animals per group). However, this shows a decrease of the sEPSP amplitudes at 10–18 months in the *App*^*NL-F/NL-F*^ mice compared with the younger cohorts. The frequency increased by, 51 ± 13% and 167 ± 8% in 1–2 months and 4–6 months aged *App*^*NL-F/NL-F*^ mice compared with control mice (*P* < 0.0001, *n =* 10 cells, *n* = 7 animals), and also reduced at 10–18 months by, 13 ± 12% of control wild-type mice (Fig. 3*A*,*F*,*G*).

In the presence of TTX (1 μM), the hyperexcitability in terms of increased action potential discharge, increased frequency and amplitudes of sEPSPs observed in AD mice was blocked, suggesting that the effect was mediated through changes in presynaptic action potential activity (Fig. 3*H*). The peak mean amplitude and frequency of mEPSPs recorded from 10 to 18 months old wild-type mice was, 0.5 ± 0.05 mV and 1.5 ± 0.34 Hz, respectively, which was not significantly different from the amplitude and frequency of mEPSPs recorded from 10 to 18 months *App*^*NL-F/NL-F*^ mice (amplitudes and frequency, 98 ± 0.3% and 95 ± 1.5% of control, respectively, *P* > 0.5, *n =* 10 cells, *n* = 5 per group). Similarly, mIPSP mean amplitude and frequency recorded in the same control animals was, 0.2 ± 0.03 mV and 1.8 ± 0.4 Hz, respectively, and also did not significantly differ from the events recorded in *App*^*NL-F/NL-F*^ mice (amplitudes and frequency, 90 ± 2.5% and 94 ± 4.5% of control, respectively, *P* > 0.5, *n =* 10 cells, *n* = 5 per group).

The overall reduction in excitation in the 10–18 months cohort was consistent with the pattern of neurodegeneration observed in the LEC of *App*^*NL-F/NL-F*^ mice compared with other cortical regions including CA1 and the neocortex as described above (Fig. 1*J*,*K*).

### Intrinsic Hyperexcitation of Principal Pyramidal Cells Correlates With Morphological Degeneration

**Figure 4.**
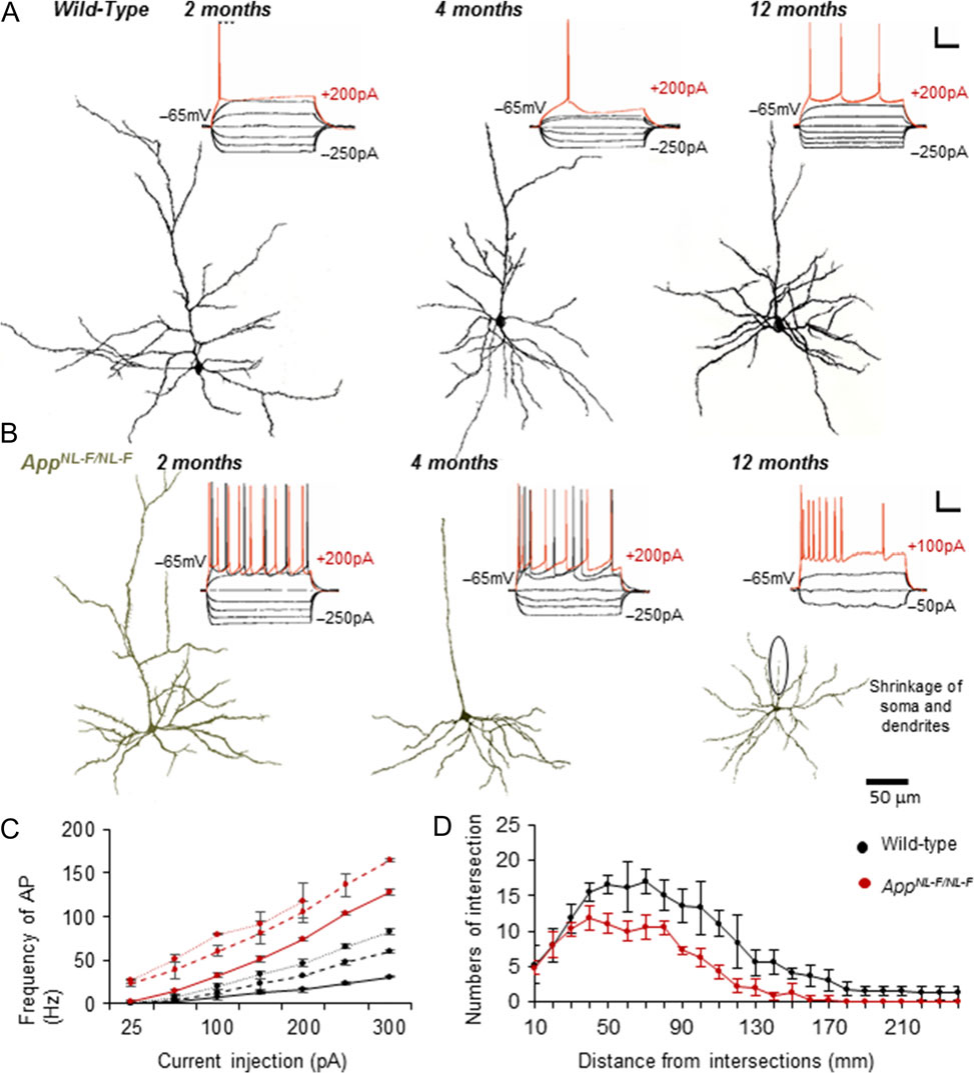
Pyramidal cell membrane hyperexcitability correlates with neurodegeneration. (*A*) and (*B*) Intrinsic membrane response of excitatory neurons of lateral entorhinal cortex (LEC) recorded in wild-type (WT) mice showed passive responses to intracellular current injection (range + 200−250 pA), which induced a single action potential with increased positive current injections (red traces). In *App*^*NL-F/NL-F*^ mice, similar currents resulted in a significant increase in action potential discharge, increased membrane resistance and increased time constants, suggesting a more excitable membrane state (*P *< 0.05, *n* = 30 neurons; 10 animals per cohort). The hyperexcitability then declined with time, resulting in impaired membrane physiology from 10 to 18 months onwards in AD mice. This was correlated with the morphological atrophy of layer 2 LEC principal cells from 4 months onward in the AD *App*^*NL-F/NL-F*^ model, (green) compared with age-matched wild-type neurons (black). Neurons were reconstructed using a light microscope and drawing tube under 100× objective. Scale refer to 20 mV and 100 ms. (*C*) Plot of action potential frequency with current injection in wild-type and age-matched App^*NL-F/NL-F*^ mice at the 3 different age cohorts studied. (*D*) The average number and length of dendrites per groups of cells were compared and found to be significantly different and Sholl analysis, was used as a measure of dendritic complexity of pyramidal cells in LEC of aged *App*^*NL-F/NL-F*^ and wild-type mice. The intersection numbers of pyramidal cells in aged *App*^*NL-F/NL-F*^ mice were significantly decreased between 40 and 240 μm from soma compared with aged wild-type mice. Results are expressed as mean ± SD (*P* < 0.05; 2-tailed Student *t*-test).

We then investigated whether the aberrant hyperexcitation related to intrinsic firing was sustained during the 3 age cohorts over time. All pyramidal cells recorded were anatomically recovered postrecording. These cells in the *App*^*NL-F/NL-F*^ mouse model at 1–2 and 4–6 months displayed greater sensitivity to intracellular current injection (Fig. 4*A*,*B*), which was very similar to previous observations made in epileptic tissue (Khan et al. 2018). This was shown by an increase in membrane resistance, reflective of a larger change in membrane voltage per current step. Further supporting this greater sensitivity, was an observed increase in the time constant, rendering neurons more excitable for the same injected positive current step. This was demonstrated from the decreased firing threshold and increased frequency of action potential discharge in the *App*^*NL-F/NL-F*^ mouse model cells compared with wild-type litter mates (Fig. 4*C*). The increase in membrane hyperactivity was apparent from the earliest time window studied, but gradually showed significant deterioration with a significant reduction in membrane resistance and time constant and the inability of the neurons to fire action potentials in the 10–18 months aged mice (Fig. 4*B*,*C*). These biophysical changes correlated with the morphological alteration of pyramidal cells in the LEC that represent progressive neurodegeneration in the *App*^*NL-F/NL-F*^ mouse model in comparison to the wild-type mice over time. This was also evidenced by the Sholl analysis used as a measure of dendritic complexity of LEC pyramidal cells in *App*^*NL-F/NL-F*^ mice compared with age matched wild-type mice, illustrated by a downwards shift in the Sholl plot (Fig. 4*D*).

### Impaired Functions of Major Inhibitory PV-Expressing Interneurons in LEC of App^NL-F/NL-F^ Mice

Over activity of the LEC in early AD suggests a loss of the inhibitory drive that controls overexcitation, which was evident from the sIPSP studies in the *App*^*NL-F/NL-F*^ mouse model. To determine the underlying mechanisms for the observed reduced inhibition, we investigated PV-expressing inhibitory interneurons. These predominantly fast-spiking neurons are the major inhibitory cells and therefore modifications of this network are likely to affect the observed imbalance between excitation and inhibition.

**Figure 5.**
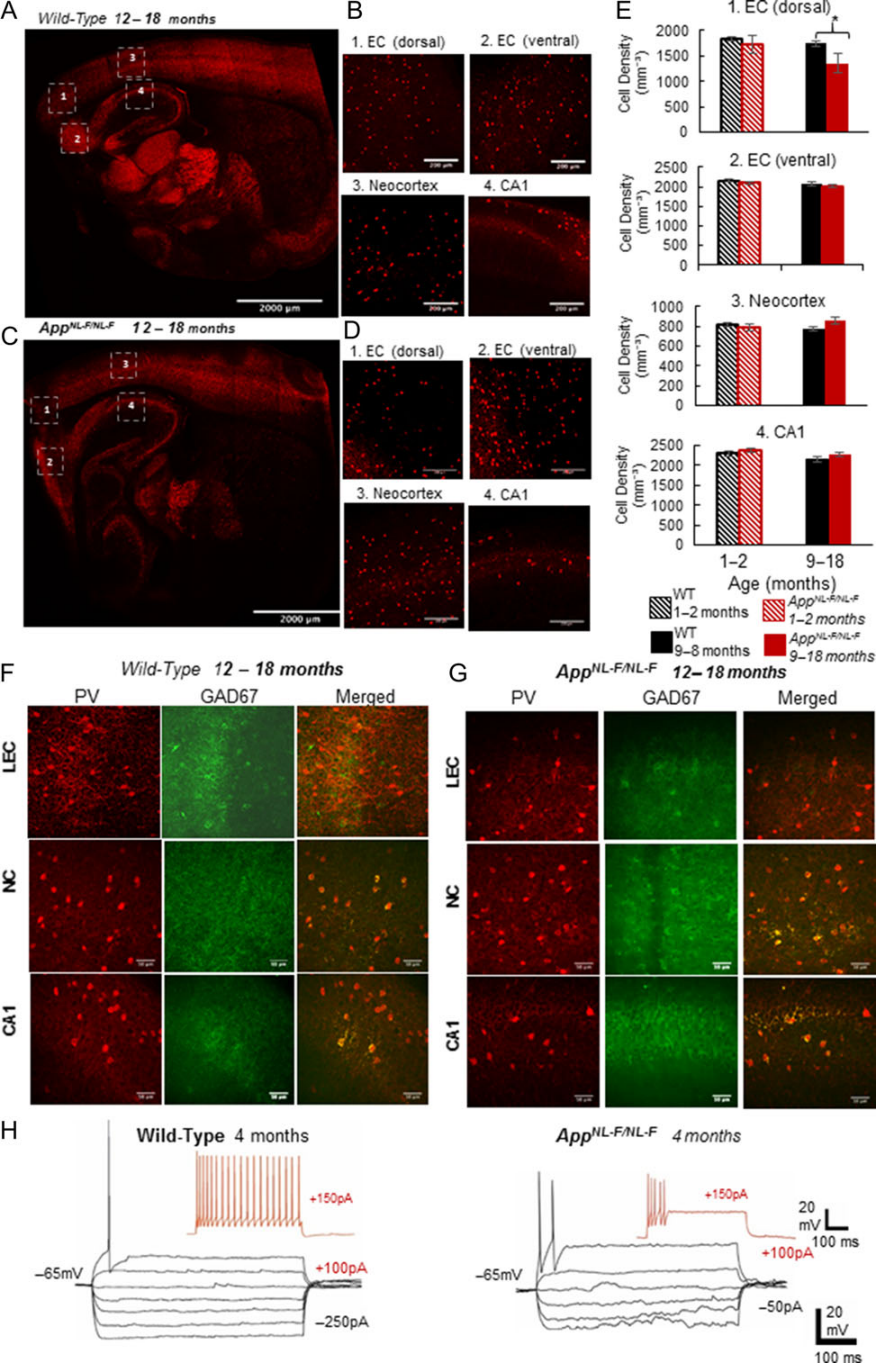
Fast-spiking parvalbumin-containing (PV) interneurons are reduced and functionally impaired in dorsal LEC in AD. Fast-spiking PV-expressing cells, a major class of interneurons that are known as the “pace makers” of the brain and responsible for regulating excitation. (*A*–*D*) Confocal microscope images from immunofluorescence labeling illustrating a significantly reduced density of PV cells in the LEC but not in neocortex or hippocampus of aged *App*^*NL-F/NL-F*^ mice (red cells, primary antibody: rabbit-anti-PV, secondary antibody: Texas red). Images (*A* and *C*) are tile scans of the whole brain section, while (*B* and *D*) are 20× magnified *Z*-stack images (20×, scale bar = 200 μm). (*E*) The bar graph shows insignificant differences in PV cell densities in WT and *App*^*NL-F/NL-F*^ mice in various cortical regions, except the dorsal LEC, suggesting that PV cells are susceptible to dysfunction in the dorsal LEC proceeding phenotypic changes of AD. PV cell counts taken from three 100 μm parasagittal brain slices of either a WT control or *App*^*NL-F/NL-F*^ brains, and counts were obtained by producing a 20× magnified *Z*-stack image at the same positions within the different cortical regions numbered from 1 to 4 on the figure (ANOVA tests performed to show statistical significance, *n* = 8 animals per age cohort). (*F*,*G*) Colocalisation experiments with PV and GAD67 (an enzyme for inhibitory neurotransmitter GABA production, primary antibody: mouse anti-GAD67, secondary antibody: FITC (green)) illustrates that less GAD67 is colocalised within PV expressed cells in aged AD mice (shown in yellow/orange fluorescence, Pearson’s correlation coefficient, *r* shown, *n* = 6 animals per cohort). This suggests that there is a decrease in the available neurotransmitter GABA in aged *App*^*NL-F/NL-F*^ brains. (*H*) Impaired intrinsic membrane properties of fast-spiking PV cells in the LEC in aged *App*^*NL-F/NL-F*^ brains.

Immunofluorescence studies displayed a significant reduction in the neuronal density of PV-expressing cells in the dorsal LEC at 10–18 months compared with age-matched wild-type counterparts (reduction of 23 ± 4%, *P* < 0.005, *n* = 8 mice per cohort). This was in contrast to a nonsignificant change in the cell densities of PV-expressing cells in the neocortex and CA1 region of the hippocampus (Fig. 5*A*–*E*). In addition, *App*^*NL-F/NL-F*^ mice at 10–18 months showed a reduction in the intensity of glutamic acid decarboxylase 67 (GAD67) within PV-expressing cells as a measure of GABA content and presynaptic function in the dorsal LEC when compared with the age-matched wild-type cohort (*P* < 0.003, Pearson’s correlation coefficient, *n* = 8 animals) (Fig. 5*F*,*G*).

These observations were accompanied by general poor health of fast-spiking interneurons recorded in the LEC, which showed a reduced action potential threshold, increased spike frequency adaption and accommodation, and inability to sustain a high frequency of firing, suggesting impaired membrane properties (Fig. 5*H*).

### GABA_A_ Receptor Allosteric Modulators Rectify Synaptic Imbalance in *App^NL-F/NL-F^* Mice

**Figure 6.**
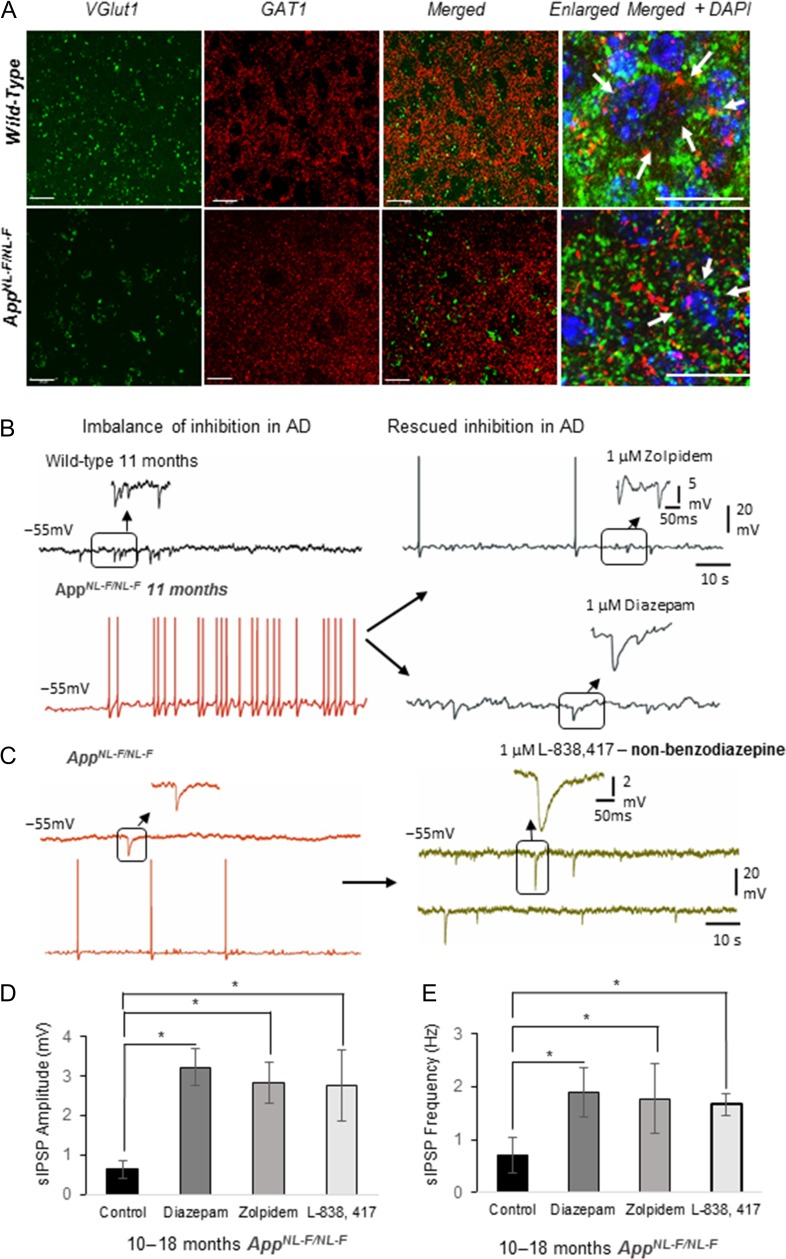
Restoration of the excitatory–inhibitory balance by GABA_A_ receptor allosteric modulators. (*A*) Immunofluorescence Z-stacks of VGlut1 (green) and GABA-transporter 1 (GAT-1, red) in entorhinal cortex in both wild-type and *App*^*NL-F/NL-F*^*mice.* Although, the levels of GAT-1 remained constant (*P* > 0.05) in both wild-type and *App*^*NL-F/NL-F*^ mouse model, the pattern of distribution of VGlut1 and GAT-1 merged, appear different in the *App*^*NL-F/NL-F*^ mice, indicated by the arrows in the enlarged merged images. (*B*,* C*) Aberrant spontaneous synaptic events recorded in principal LEC neurons in wild-type and App^*NL-F/NL-F*^ mice restored by bath-application of GABA_A_ receptor allosteric modulators diazepam, α1 subunit containing GABA_A_R, zolpidem or α2 and α3 subunit containing GABA_A_ receptor (nonbenzodiazepine), L-838, 417. (*D*) Bar graphs to illustrate the change in the amplitude and frequency of sIPSPs with bath-application of diazepam, zolpidem or L-383,417, (*P < 0.05; *n* = 4, 10–18 months old animals per cohort and *n* = 6 cells per cohort).

It has been established that pyramidal cell somata and axon initial segments receive only inhibitory synapses from axon terminals of PV-expressing interneurons. To determine whether perisomatic inhibitory terminals were still active in the *App*^*NL-F/NL-F*^ mouse model, we performed immunofluorescence studies using anti-GABA transporter 1 (GAT1) (Fig. 6*A*) localized with the antibody against vesicular glutamate transporter 1 (VGluT1), that marks terminals of the major excitatory neurotransmitter glutamate pathways. Thus, the pattern and the extent of colocalisation of GAT1 with VGluT1 will reveal whether perisomatic PV axon terminals innervate pyramidal cells to the same extent in late AD as in control animals. The pattern of the punctate structures of VGluT1 were sparse but appeared as more intense clustered immunoreactivity in the aged *App*^*NL-F/NL-F*^ mice. This GABAergic marker was found in puncta present in the periphery of cell bodies, which seemed to be reduced in distribution around DAPI stained somata in the *App*^*NL-F/NL-F*^ mice at 10–18 months. However, there was some preservation of GAT1-positive puncta around the neuronal somata despite the apparent reduction in the functionality of PV cells (as indicated by GAD67 and PV firing properties), therefore, we investigated whether pharmacological manipulations of the existing GABAergic terminals could rectify the observed synaptic imbalance and hyperexcitation. Following bath-application of the broad spectrum GABA_A_ receptor allosteric modulator, diazepam (1 μM) (Fig. 6*B*), the hyperexcitability of pyramidal cells in the LEC was reduced by 60 ± 6.5% (*P* < 0.05, *n* = 6 cell, 4 animals at 6 months, and 10–18 months), and furthermore, the aberrant inhibition was restored, which resulted in an increase in amplitude and frequency of sIPSPs (increase of 330 ± 10% and 126 ± 9.5%, in amplitude and frequency, respectively, *P* < 0.05, *n* = 6 cell, 4 animals) which was consistent with previous studies (Busche et al. 2008). However, diazepam as a potential therapeutic target for AD is controversial (Nicholson et al. 2018) thus, we further investigated specific GABA_A_ receptor allosteric modulators including zolpidem (1 μM), a modulator of α1-containing GABA_A_ receptors, and L-838,417 (0.5 μM), a selective modulator of α2- and α3- containing GABA_A_Rs, classed as a nonbenzodiazepine anxiolytic (Atack 2011). Like diazepam, both, zolpidem (Fig. 6*B*) and L-838,417 restored sIPSP amplitudes, which increased by 273 ± 32% and 246 ± 13% of control values, respectively (*P* < 0.05, *n* = 4 animals) (Fig. 6*C*). The sIPSP frequency also increased with zolpidem and L-838,417 by 97 ± 36% and 85 ± 13%, respectively (*P* < 0.05, *n* = 4 animals), suggesting restoration of synaptic excitatory–inhibitory imbalance (Fig. 6*D*,*E*). However, bath-application of the GABA_A_ receptor antagonist gabazine (50 μM) increased the sEPSP amplitude and frequency and decreased sIPSP parameters comparable to control untreated levels. The overall findings after bath application of diazepam, zolpidem and L-838,417, is that the aberrant, depleted inhibitory synaptic events observed in 10–18 months old *App*^*NL-F/NL-F*^ mice was “normalized” and these events were similar in amplitude and frequency to the inhibitory events recorded in the age-matched wild-type mice. These changes with diazepam and zolpidem were also consistent with previous studies using healthy control rodents (Ali and Thomson 2008).

## Discussion

Using the first AD knock-in mouse model (*App*^*NL-F/NL-F*^), which faithfully recapitulates disease progression in AD patients as shown by molecular studies (Saito et al. 2014; Sasaguri et al. 2017), we explored progressive changes in synaptic mechanisms underlying AD pathology in the LEC with the following 3 key findings: firstly, we showed initial mechanistic synaptic dysfunction including a persistent hyperexcitation of pyramidal cell membrane properties and a diminished synaptic excitatory–inhibitory balance correlated with a reduction in canonical Wnt signaling activity in the LEC. Secondly, we identified that the impaired excitatory–inhibitory balance primarily originated from a decreased cellular distribution and hypoactivity of GABAergic function of PV interneurons in the LEC. Finally, we showed that the impaired synaptic imbalance was restored by applying specific GABA_A_ receptors allosteric modulators, delineating future early stage therapeutic targets to prevent or halt mechanisms of synaptic dysfunction leading to neurodegeneration associated with AD.

The entorhinal cortex is the most vulnerable cortical region affected during early stages of AD, and our results show that the majority of principal cells are hyperactive before the presence of the hallmarks of disease, neuroinflammation or accumulation of Aβ plaques. However, others have suggested that early hyperexcited neurons in cortical regions are associated with amyloid plaques in a double transgenic (App23, PS45) AD mouse model (Busche et al. 2008).

Initial triggers of the observed hyperactivity and pathogenesis seem to be a combination of synaptic and molecular dysregulation. The results from our TTX experiments suggest a strong network-driven component that contributes to the sustained hyperexcitability, as blocking action potential discharge eliminated synaptic hyperactivity. The dysregulated network probably combines excitatory and inhibitory components. We suggest the network-driven hyperexcitability is related to changes in the fundamental inhibitory PV microcircuitry, which was significantly reduced in density, firing properties and the capacity to produce the neurotransmitter GABA in the LEC. These factors alone (due to a loss of the inhibitory drive) or in combination with other dysfunctional dis-inhibitory interneurons in the *App*^*NL-F/NL-F*^ mouse model (Shi et al., manuscript under review), could potentially trigger synaptic imbalance and hyperactivity of the pyramidal cells in the LEC in early AD. The LEC was unique in this change, since we report unchanged PV cell densities in other cortical regions, including the dorsal entorhinal cortex, neocortex and hippocampus in late phenotypical expression of AD in *App*^*NL-F/NL-F*^ mice, although a general loss of colocalized PV with GAD67 was consistent in all cortical regions studied, suggesting a reduction of the synthesis of GABA neurotransmitter present within cell bodies and therefore inhibitory function. Similarly, we observed a reduction in GAT-1 expression at GABAergic somatic terminals, which we propose is due to PV cells since it is well established that PV cells make only proximal postsynaptic contacts (Kawaguchi et al. 1987). Others have suggested that a direct excitatory innervation of CA1 PV cells is lost due to principal cell death in the entorhinal cortex (Yang et al. 2018a,b). Whether similar mechanisms trigger the loss of LEC PV cells in the LEC needs further investigation.

The factors that cause the initial reduction in inhibition could be related to observations showing that most diminished GABA terminals are found adjacent to Aβ plaques suggesting that Aβ plaque accumulation directly initiates cellular dysfunction in patients affected by AD (Garcia-Marin et al. 2009). However, PV is a calcium binding protein that is thought to have a buffering capacity and prevents oxidative stress, which makes these cells resilient to neurodegeneration. Thus factors such as the impaired, hypoactive intrinsic properties of PV cells as we observed, could be directly related to the initial insults. Similarly, others have also reported hypoactivity of PV cell membrane properties (Zhang et al. 2016).

In line with the electrophysiological and microscopic observations, the reduction in Wnt signaling activity was first observed in the entorhinal cortex, the region first affected in AD. The magnitude of the decrease in signaling activity remained similar in the entorhinal cortex from 1 to 18 months but increased over time throughout the remaining cortex, illustrating a direct correlation between canonical Wnt signaling activity and progress of AD pathogenesis. At this stage it is clear that Wnt signaling dysregulation occurs prior to Aβ pathology and gliosis but unclear if it is the result or the cause of the early observed synaptic dysfunction. The observed early onset of pyramidal cell hyperactivity might be a manifestation of disrupted synaptic mechanisms mediated by PV network function originating in the LEC and likely amplified by canonical Wnt signaling dysfunction. However, it is also important to note that Wnt signaling is known to be crucial during brain development including axonal outgrowth and synapse formation. In addition, evidence for the relevance of Wnt signaling for synaptic maintenance and function throughout life is accumulating (Inestrosa and Arenas 2010; Jensen et al. 2012; Park and Shen 2012; Dickins and Salinas 2013; Oliva et al. 2013a,b). Therefore, dysregulation of canonical Wnt signaling possibly caused by mutant APP gene expression during development might cause subtle neuronal changes, resulting in an increased vulnerability to neurodegenerative insults that becomes evident during aging (Zhou et al. 2012; Purro et al. 2012, 2014; Tapia-Rojas et al. 2016; Elliott et al. 2018; Tapia-Rojas and Inestrosa 2018a,b). For example, knockout of the specific canonical Wnt signaling co-receptor Lrp6 leads to age related structural and functional synaptic changes in wild type mice and accelerates pathogenic changes in AD mouse models (Liu et al. 2014). Therefore, changes observed in the APP^NL-F/NL-F^ animals might be partially mimicked by knockout or knockdown of canonical Wnt signaling components such as Lrp6 in wild type animals. *Vice versa* treatment with stimulators of canonical Wnt signaling such as Wnt3a ligand or DKK1 inhibitors might alleviate some of the observed neuronal changes in the *APP*^*NL-F/NL-F*^ animals (Alvarez et al. 2004; Toledo and Inestrosa 2010; Fiorentini et al. 2010; Harvey and Marchetti 2014; Yi et al. 2014; Parr et al. 2015; Marzo et al. 2016; Jin et al. 2017, Huang et al. 2018).

Consistent with the observed synaptic imbalance, there was a notable change in the intrinsic membrane properties rendering pyramidal cells in the *App*^*NL-F/NL-F*^ mouse model more excitable compared with age matched wild-type mice. This membrane hyperactivity could be related to pro-inflammatory mediators, such as cytokines, reactive oxygen species and free radicals to name a few, released from the activated astrocytes and glial cells, which themselves have been shown to be altered morphologically (Olabarria et al. 2010; Rodriguez et al. 2010). Astrocytes regulate the microenvironment by providing K^+^ ion homeostasis for excitable membranes, and their reduced function could lead to an accumulation of extracellular K^+^ released from neurons (Verkhratsky 2010), resulting in a more depolarized membrane potential, rendering cells more excitable. These hyperactive membrane properties become weak and diminished with age, which is probably due alterations and down regulation of leak conductance responsible for generating the intrinsic firing of these cells. Moreover, hyperexcitation of neuronal populations can lead to excitotoxicity contributing to neurodegeneration, as shown in our aged *App*^*NL-F/NL-F*^ mice that were associated with morphological atrophy of LEC pyramidal cells and the gradual decline in the density of principal cells as shown by the CAMKII-α labeling experiments. These cellular properties observed in vitro correlate with the behavioral abnormalities of this AD mouse model detected from 10 months onwards (Masuda et al. 2016).

Dysfunctional synaptic activity has been shown to promote the spread of Aβ (Yamamoto et al. 2015); this is further supported by the largest accumulation of Aβ plaques in post-phenotypic *App*^*NL-F/NL-F*^ mice found at the boundary between the neocortex and the dorsal entorhinal cortex, suggesting that the initial hyperexcitation observed in the entorhinal cortex promotes the propagation of Aβ. The observed aberrant function of the LEC is consistent with preclinical human fMRI studies that have also shown pathology to be initiated in the LEC (Khan et al. 2014). Therefore, it would be beneficial to understand whether such mechanisms could be normalized, as it may prevent the propagation of the disease. To normalize the impaired aberrant excitatory–inhibitory imbalance, we pharmacologically targeted the preserved GABAergic terminals in the aged *App*^*NL-F/NL-F*^ model and our results are promising. Three different types of GABA_A_ receptor allosteric modulators, diazepam, zolpidem and L-838,417, rectified the aberrant synaptic excitatory–inhibitory imbalance in our AD model by enhancing the amplitudes and frequency of inhibitory effects. We suggest that both, the increase in sIPSP amplitudes and frequency with these allosteric modulators are due to postsynaptic effects related to the enhanced affinity for GABA at the receptors, as well as an increase in the frequency of the opening times of the receptor ion channels. Hence, our observations support the idea of a reduced GABAergic network primarily contributing to the observed hyperexcitability of principal cells. Zolpidem is specifically known to target α1 containing GABA_A_ receptors, while L-838,417 has been shown primarily to activate α2 and α3 subunit-containing GABA_A_ receptors, (McKernan et al. 2000; Mathiasen et al. 2007; Ujfalussy et al. 2007), which are subtypes of GABA_A_ receptors shown to be associated with postsynaptic domains targeted by PV interneurons (Ali and Thomson 2008), and with little sedative or amnestic effects that are associated with α1 subtypes (McKernan et al. 2000). Others have also shown that AD associated insults to cortical regions can be restored or intercepted with either pharmacological agents (Busche et al. 2008; Busche and Konnerth 2016), or by optogenetic experiments inducing specific oscillatory states such as gamma (Iaccarino et al. 2016; Nakazono et al. 2017) or theta oscillations (Yang et al. 2018a,b).

In summary, AD is a multifactorial disease, and the interplay between the molecular and cellular mechanisms probably cause an iterative remodeling of cellular pathways and re-distribution of the synaptic activity during the pathogenesis of the disease. In this study, we report a combination of early stage molecular and synaptic mechanistic dysfunction that contributes to the disease pathogenesis. Canonical Wnt signaling changes in the *App* knock-in mice are observed early on in the AD disease process in areas typically also affected in AD patients and exacerbate further over time. These changes are not observed in the cerebellum, a brain area mostly spared during AD pathogenesis. Our data suggest impaired synaptic excitatory–inhibitory inputs in pyramidal cells of *App*^*NL-F/NL-F*^ mice from early stages of the disease, preceding the typical hallmarks of AD, i.e., before the signs of neuroinflammation and Aβ plaque formation in the LEC, which is also corroborated by recent study using rTg4510 transgenic mouse line studying tauopthay (Jackson et al. 2017). In the present study, we suggest that the synaptic imbalance is associated with a decrement in PV interneuron density and function specific to the dorsal entorhinal cortex, which plays a vital function in the storage of episodic and long-term memory alongside the maintenance of crucial cognitive functions in the murine brain.

In conclusion, this novel study provides a deeper understanding of the neuronal networks affected in AD which can form the basis for further mechanistic studies. Further investigation of whether early treatment preceding AD hallmarks with specific targeted GABA_A_ receptors modulators halts the neurodegeneration could lead to novel therapeutic intervention and assist in future developments of novel targeted therapies to delay, halt or prevent memory deficits associated with AD dementia.

## Supplementary Material

Supplementary Data
