# Supplementary material for: Aberrant Excitatory–Inhibitory Synaptic Mechanisms in Entorhinal Cortex Microcircuits During the Pathogenesis of Alzheimer’s Disease
Source: Cereb Cortex. 2019 Feb 15;29(4):1834–50. doi: 10.1093/cercor/bhz016 (PMC6418384; doi:10.1093/cercor/bhz016)
Supplement: Supplementary Data [file bhz016_supplementary_materials.zip › bhz016_Petrache_et_al_AD_Wnt_Supp_legends.pdf]

Supplementary Figures 1-7***. Full membrane and antibody detection for shown western blot images in figure 2 - Wnt signalling analysis of wild-type and App$^{NL\text{-}F/NL\text{-}F}$ knock-in mice***

All figures show on the left-hand side the membrane with indication of the used protein marker and on the right-hand side the full membrane with the signal. The cutting region for figure 2 is indicated with a black surrounding of the specific bands. An arrow is pointing to the specific bands in figure S1. The membranes are correlated as following: figure S1 belongs to figure 2A, figure S2 belongs to figure 2D (left), figure S3 belongs to figure 2D (middle), figure S4 belongs to figure 2D (right), figure S5 belongs to figure 2E (left), figure S6 belongs to figure 2E (middle), figure S7 belongs to figure 2E (right).
