# Supplementary figures and images for: Aberrant Excitatory–Inhibitory Synaptic Mechanisms in Entorhinal Cortex Microcircuits During the Pathogenesis of Alzheimer’s Disease
Source: Cereb Cortex. 2019 Feb 15;29(4):1834–50. doi: 10.1093/cercor/bhz016 (PMC6418384; doi:10.1093/cercor/bhz016)

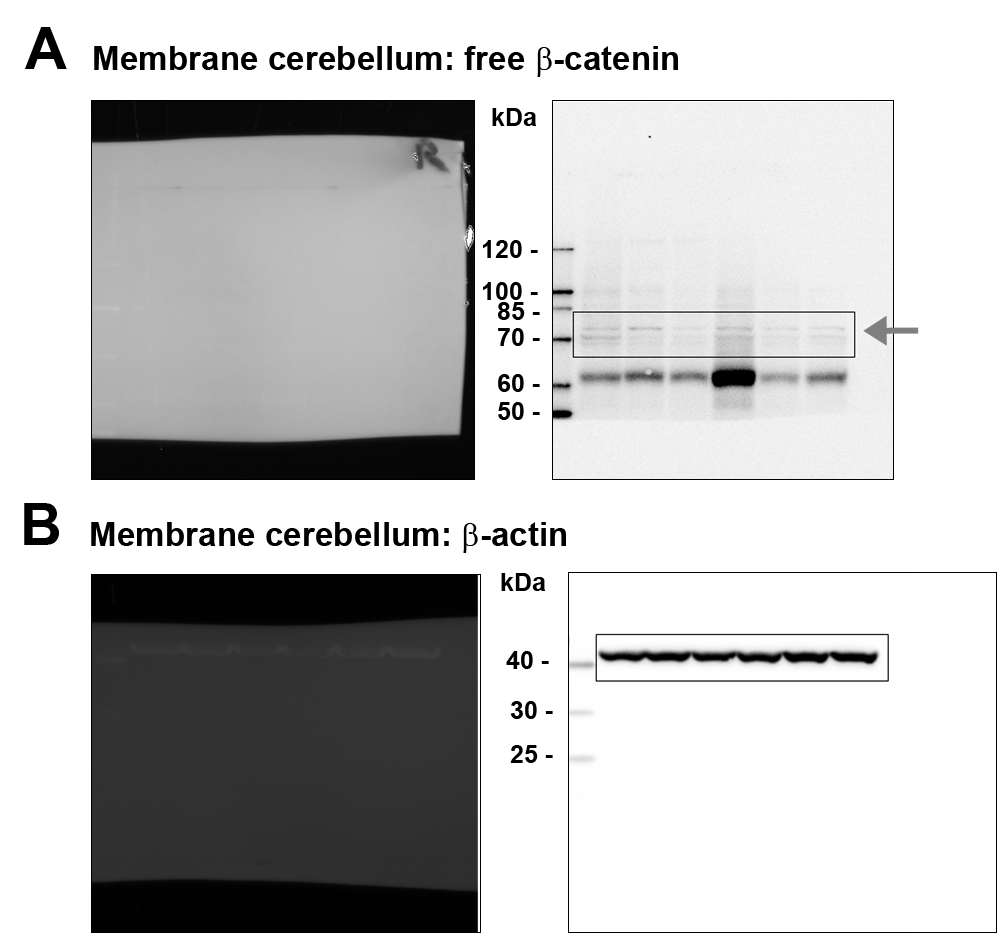

Supplement: Supplementary Data [file bhz016_supplementary_materials.zip › bhz016_Supplement_figure_1_new_1118.png]

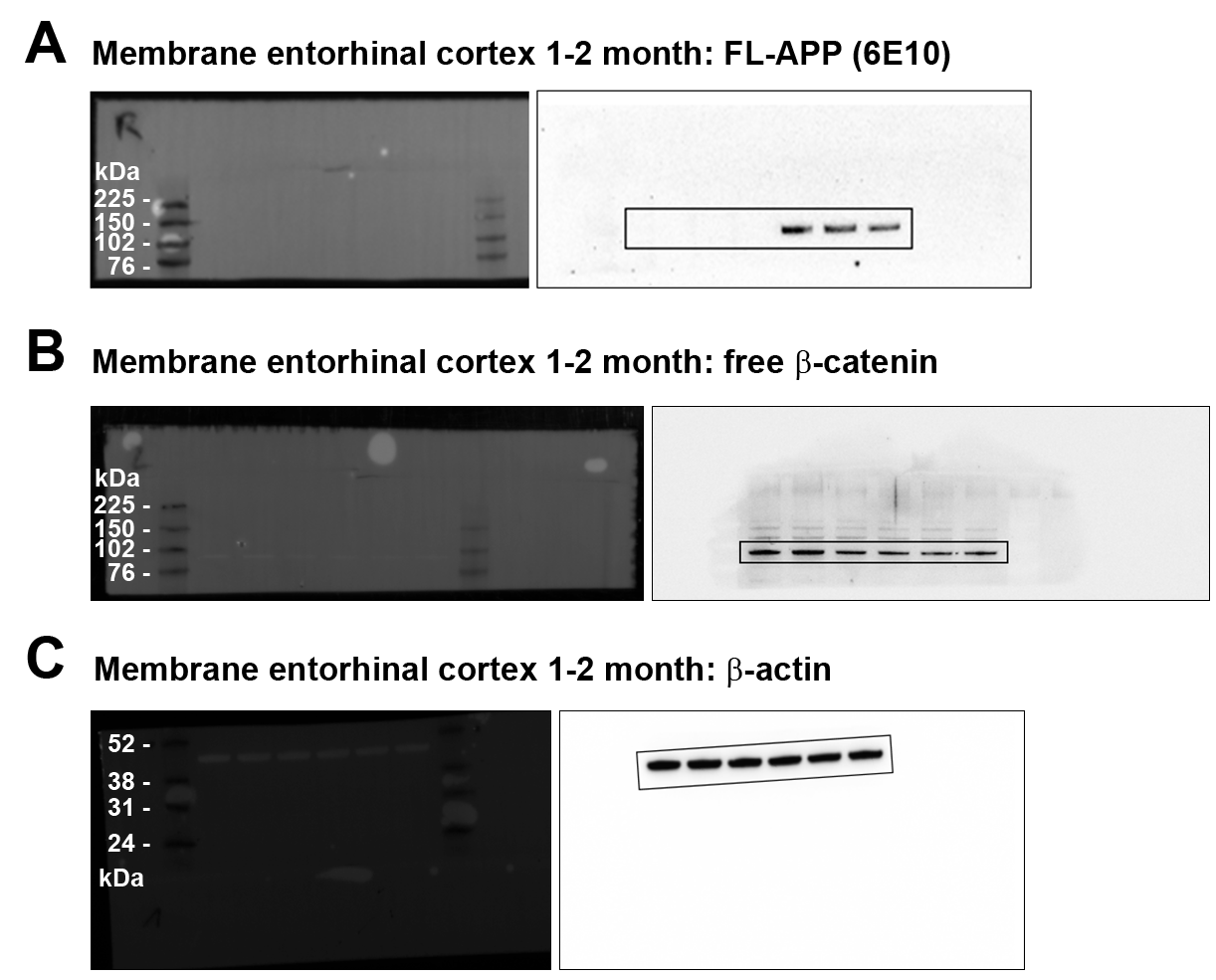

Supplement: Supplementary Data [file bhz016_supplementary_materials.zip › bhz016_Supplement_figure_2_new_1118.png]

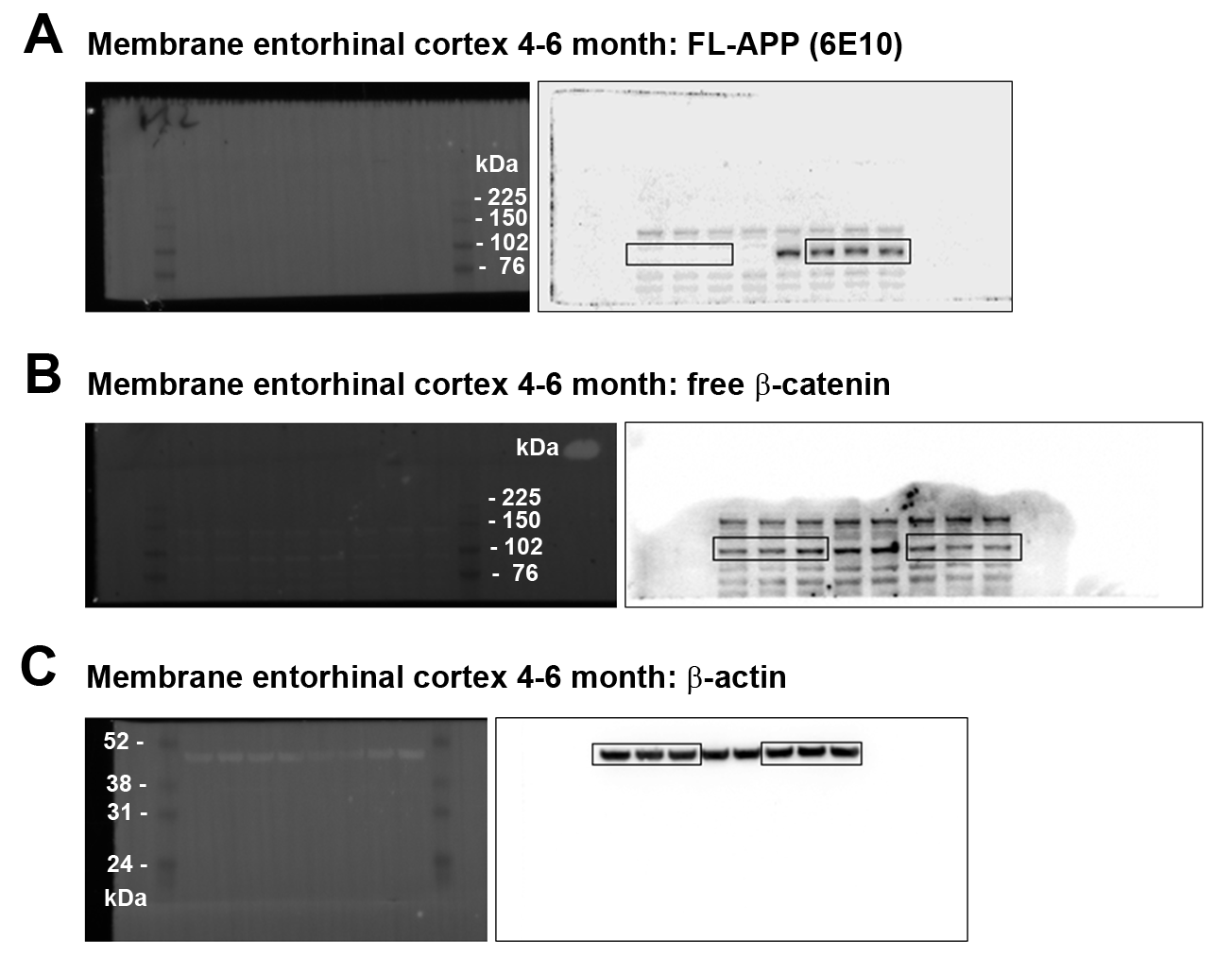

Supplement: Supplementary Data [file bhz016_supplementary_materials.zip › bhz016_Supplement_figure_3_new_1118.png]

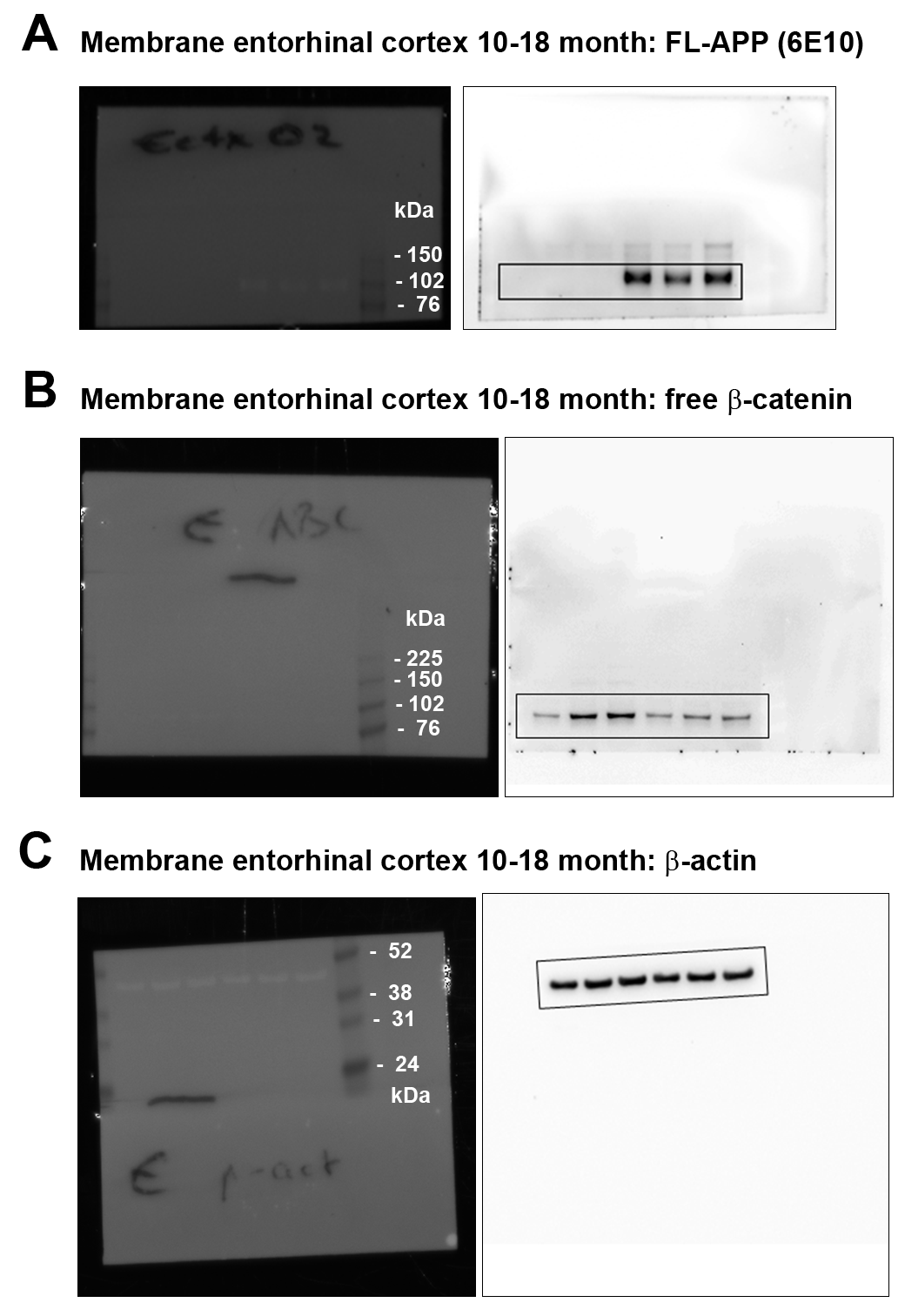

Supplement: Supplementary Data [file bhz016_supplementary_materials.zip › bhz016_Supplement_figure_4_new_1118.png]

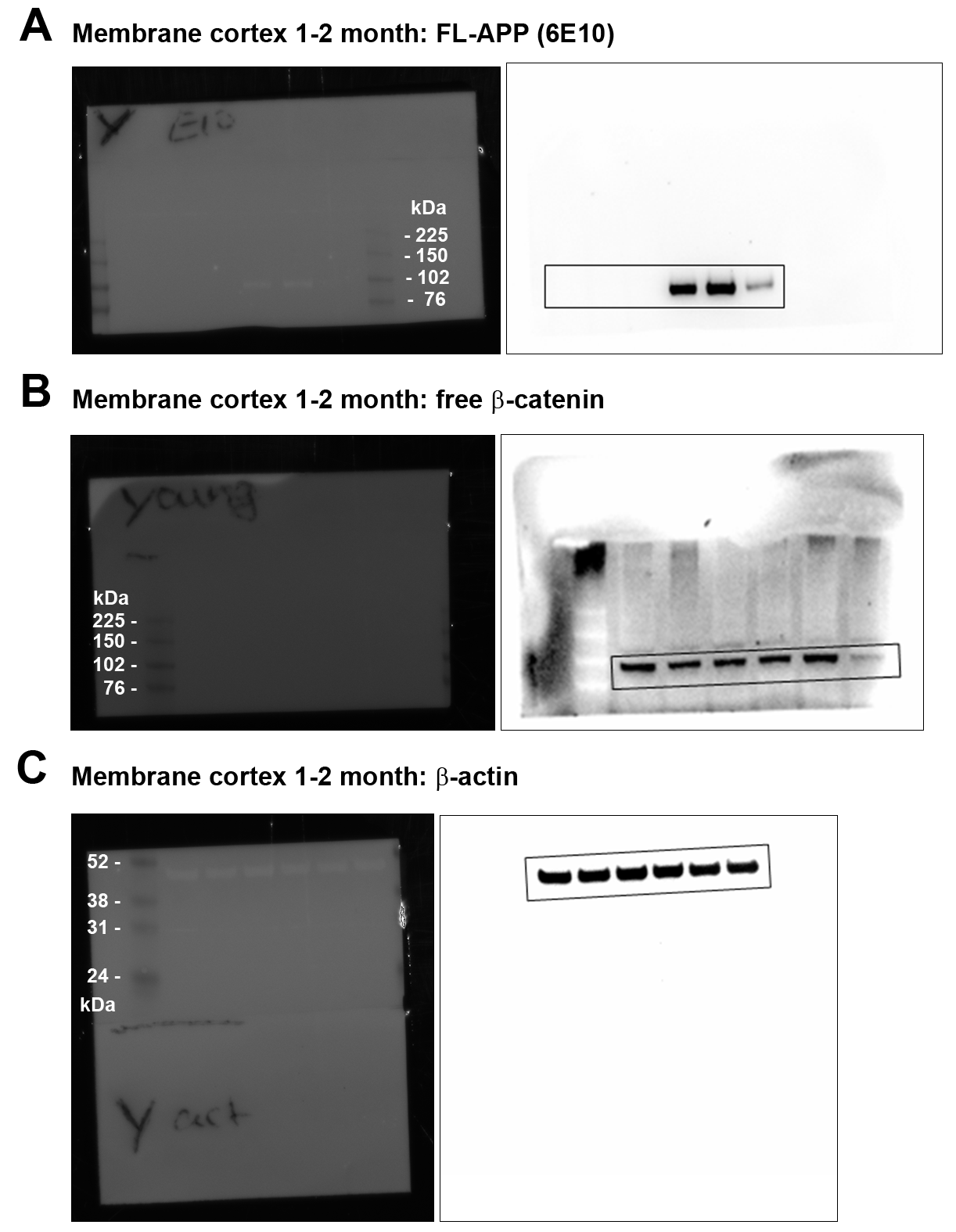

Supplement: Supplementary Data [file bhz016_supplementary_materials.zip › bhz016_Supplement_figure_5_new_1118.png]

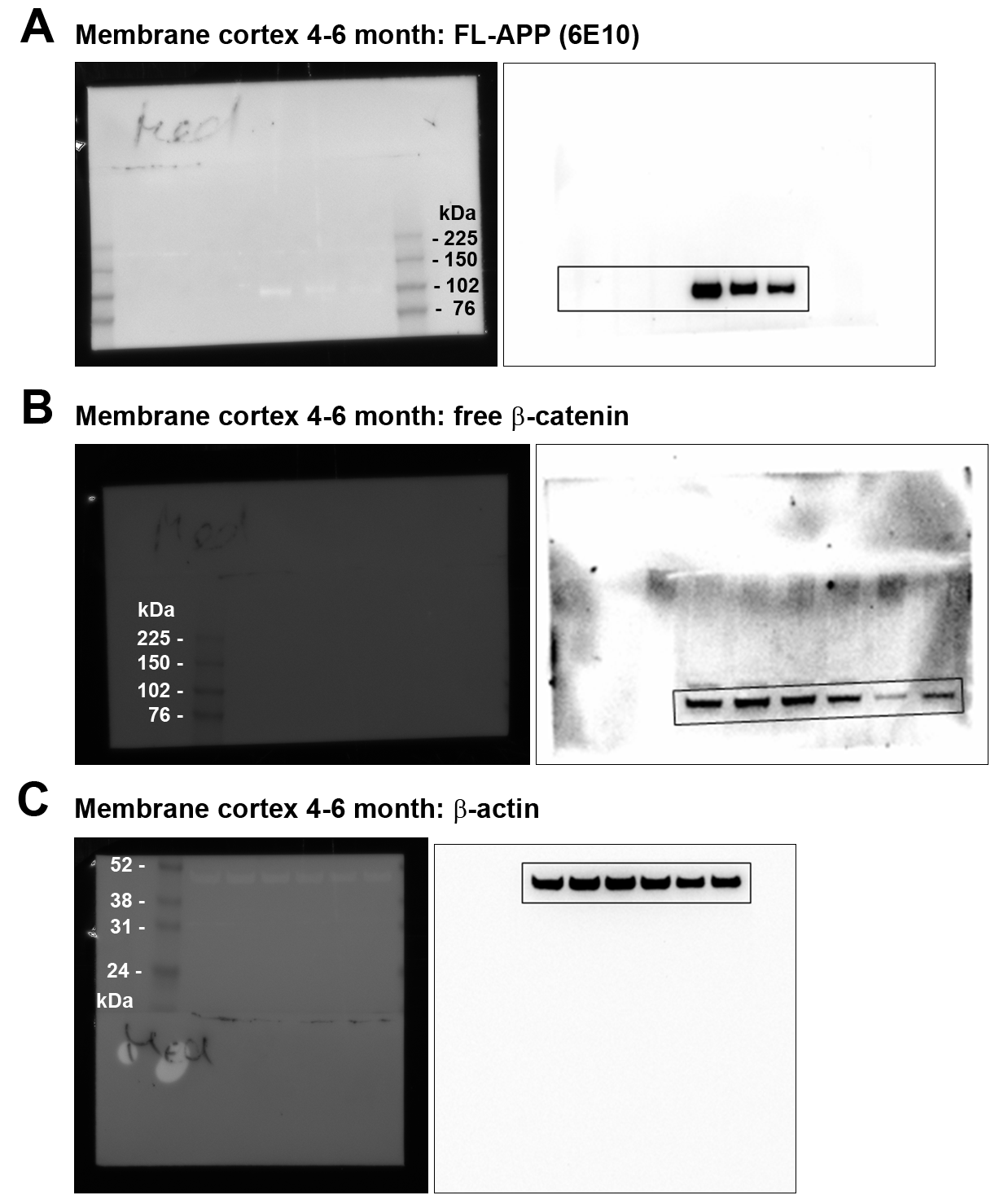

Supplement: Supplementary Data [file bhz016_supplementary_materials.zip › bhz016_Supplement_figure_6_new_1118.png]

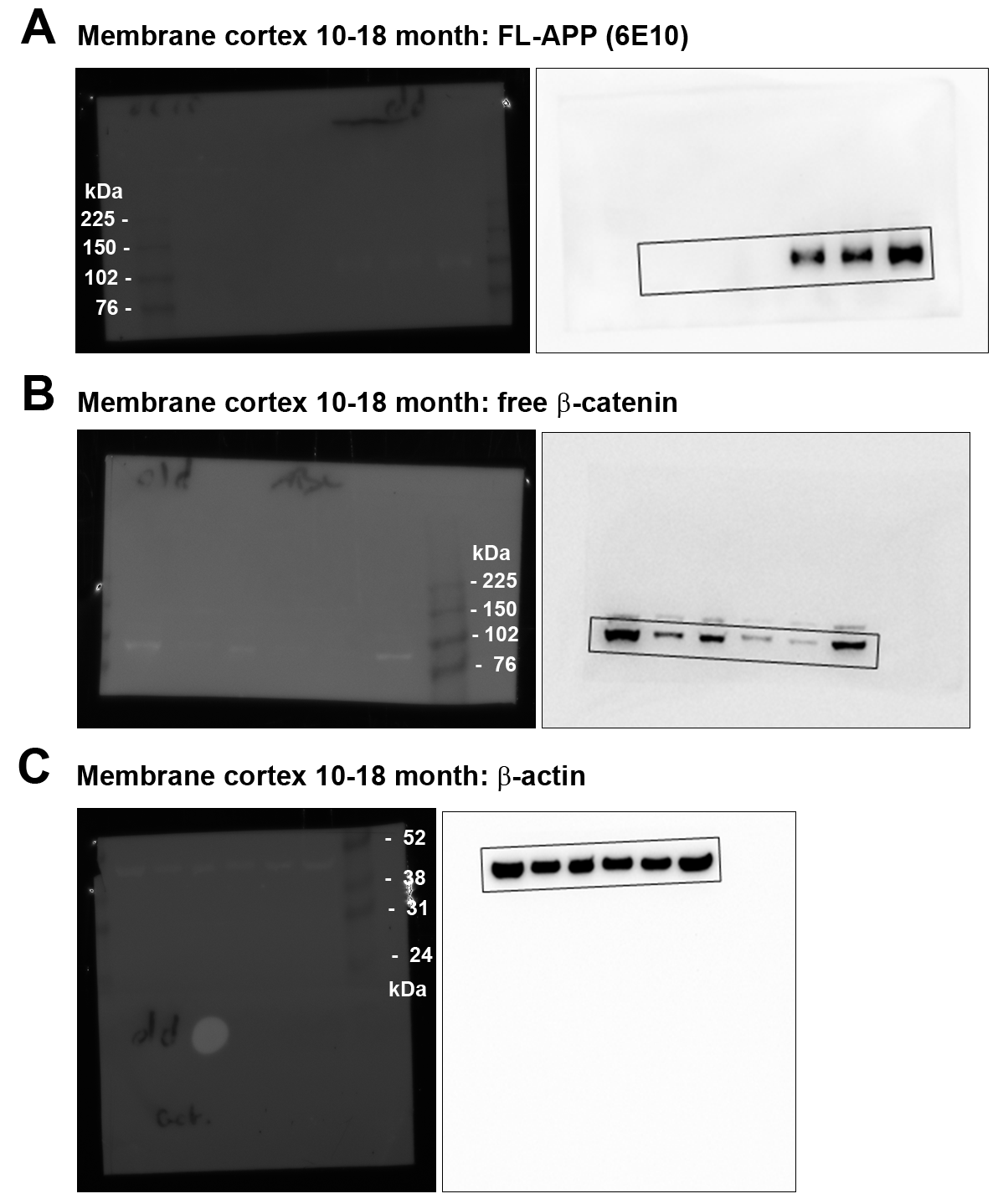

Supplement: Supplementary Data [file bhz016_supplementary_materials.zip › bhz016_Supplement_figure_7_new_1118.png]
